# Environmental Enrichment Prevents Transcriptional Disturbances Induced by Alpha-Synuclein Overexpression

**DOI:** 10.3389/fncel.2018.00112

**Published:** 2018-04-24

**Authors:** Zinah Wassouf, Thomas Hentrich, Sebastian Samer, Carola Rotermund, Philipp J. Kahle, Ingrid Ehrlich, Olaf Riess, Nicolas Casadei, Julia M. Schulze-Hentrich

**Affiliations:** ^1^Institute of Medical Genetics and Applied Genomics, University of Tübingen, Tübingen, Germany; ^2^Centre for Integrative Neuroscience, University of Tübingen, Tübingen, Germany; ^3^German Center for Neurodegenerative Diseases, Tübingen, Germany; ^4^Hertie Institute for Clinical Brain Research, University of Tübingen, Tübingen, Germany; ^5^Department of Neurobiology, IBBS, University of Stuttgart, Stuttgart, Germany

**Keywords:** alpha-synuclein, enriched environment, Parkinson's disease, gene-environment interaction, immediate early genes

## Abstract

Onset and progression of neurodegenerative disorders, including synucleinopathies such as Parkinson's disease, have been associated with various environmental factors. A highly compelling association from a therapeutic point of view has been found between a physically active lifestyle and a significantly reduced risk for Parkinson's disease. Mimicking such conditions in animal models by promoting physical activity, social interactions, and novel surroundings yields in a so-called enriched environment known to enhance adult neurogenesis, increase synaptic plasticity, and decelerate neuronal loss. Yet, the genes that connect beneficial environmental cues to the genome and delay disease-related symptoms have remained largely unclear. To identify such mediator genes, we used a 2 × 2 factorial design opposing genotype and environment. Specifically, we compared wildtype to transgenic mice overexpressing human *SNCA*, a key gene in synucleinopathies encoding alpha-synuclein, and housed them in a standard and enriched environment from weaning to 12 months of age before profiling their hippocampal transcriptome using RNA-sequencing. Under standard environmental conditions, differentially expressed genes were overrepresented for calcium ion binding, membrane, synapse, and other Gene Ontology terms previously linked to alpha-synuclein biology. Upregulated genes were significantly enriched for genes attributed to astrocytes, microglia, and oligodendrocytes. These disturbances in gene activity were accompanied by reduced levels of several presynaptic proteins and the immediate early genes EGR1 and NURR1. Intriguingly, housing transgenic animals in the enriched environment prevented most of these perturbations in gene activity. In addition, a sustained activation specifically in transgenic animals housed in enriched conditions was observed for several immediate early genes including *Egr1, Nr4a2*/*Nurr1, Arc*, and *Homer1a*. These findings suggest a compensatory mechanism through an enriched environment-activated immediate early gene network that prevented most disturbances induced by alpha-synuclein overexpression. This regulatory framework might harbor attractive targets for novel therapeutic approaches that mimic beneficial environmental stimuli.

## Introduction

Accumulation of misfolded alpha-synuclein in intracellular inclusions known as Lewy bodies is the pathological hallmark of Parkinson's disease, dementia with Lewy bodies, and multiple system atrophy, collectively referred to as synucleinopathies (Spillantini et al., 1997). Data from genetic studies highlight the role of alpha-synuclein as rare point mutations in *SNCA* (Eriksen et al., 2003) and genomic multiplications of that locus (Chartier-Harlin et al., 2004) are linked to familial forms of Parkinson's disease in a gene dose-dependent manner. Further, genome-wide association studies and meta-analyses of their data (Simón-Sánchez et al., 2009) revealed polymorphisms in *SNCA* as strong susceptibility factors for sporadic Parkinson's disease. Genetic defects, however, account for only a small fraction of Parkinson's disease cases, the majority of patients does not have a straightforward genetic *SNCA* predisposition (Houlden and Singleton, 2012).

Adding to the etiological complexity of synucleinopathies, various environmental factors have been associated with disease onset and progression without much insight yet as to how these factors are integrated into the regulatory program and modulate gene activity (Ascherio and Schwarzschild, 2016). Recent results from meta-analyses highlight physical activity, specifically a medium level of exercise, to have one of the most significant associations (Bellou et al., 2016) with a reduced risk of developing Parkinson's disease (Yang et al., 2015). Mimicking physical activity combined with cognitive stimulation in the laboratory can be achieved by enriched environments (EE) that augment the physical and social complexity of living conditions for rodents (Nithianantharajah and Hannan, 2006). EEs have been shown to improve behavioral as well as cognitive performance and to decelerate neuronal loss in several neurodegenerative disorders including Parkinson's, Huntington, and Alzheimer's disease (Nithianantharajah and Hannan, 2006). In Parkinson's disease, EE was proven to ameliorate behavioral impairments and attenuate neuronal and glial insults in both MPTP- (Bezard et al., 2003; Faherty et al., 2005; Goldberg et al., 2011; Klaissle et al., 2012) and 6-OHDA-induced (Jadavji et al., 2006; Steiner et al., 2006; Anastasia et al., 2009; Jungling et al., 2017) animal models. However, the underlying genes that connect beneficial environmental cues to the genome and delay disease-related symptoms have remained largely enigmatic.

To identify such genes, we cross-compared transgenic mice overexpressing human *SNCA* with wildtype animals and the effect of a long-term environmental enrichment, from weaning to 12 months of age, with standard housing conditions, by using a factorial design of experiments that assessed the effects of genotype, environment, and their interaction. Employing RNA-sequencing, we profiled gene expression in the hippocampus as this brain region represents (i) an area linked to early, non-motor characteristics of alpha-synuclein pathology such as cognitive deficits (e.g., memory retrieval and decision making) and behavioral changes (e.g., depression and anxiety) (Chaudhuri et al., 2006), and (ii) a central brain hub for integrating sensory information from external stimuli (Kempermann et al., 1997). Under standard environmental conditions, *SNCA* overexpression perturbed a diverse set of genes attributed to distinct cell types and overrepresented for Gene Ontology terms previously related to alpha-synuclein biology. These disturbances in gene activity were paralleled by reduced levels of presynaptic proteins and the immediate early genes (IEGs) EGR1 and NURR1. Strikingly, these disturbances were largely prevented when transgenic mice were housed in the EE. Expression analyses revealed a group of genes responding to the enriched environment specifically in transgenic animals, among them several IEGs including *Erg1, Nr4a2*/*Nurr1, Arc*, and *Homer1a*, suggesting their sustained activation and associated regulatory framework provided a means to compensate for perturbations induced by *SNCA* overexpression.

## Materials and methods

### Generation of transgenic mice

A *Bacterial Artificial Chromosome* (BAC) construct comprising a fused PAC AF163864 and BAC AC097478 which contains the entire human *SNCA* gene locus with 28 kb 5′- and 50 kb 3′-flanking regions was used to generate the transgenic mouse model (Yamakado et al., 2012). C57BL/6N mice from Charles River were used to generate the transgenic animals as described previously (Nuber et al., 2013). *High Pure PCR Template Preparation* kit (Roche) was used to isolate DNA from ear biopsies for genotyping (see Supplementary Table 1 for primers sequences). Only homozygous mice were used in this study, which was confirmed using quantitative real-time PCR performed on a *LightCycler 480* (Roche). Genotyping was performed after weaning and confirmed when animals were sacrificed.

### Study design and environmental enrichment

After weaning, wildtype (WT) and transgenic (TG) animals were randomly assigned to either the standard (SE) or the enriched (EE) environment. As aggressive behavior has been reported to occur between male mice housed in a long-term enriched environment (Marashi et al., 2003) only female mice were used. In SE, groups of three to four female mice were housed in standard cages (365 × 207 × 140 mm, *Typ II long*) with normal light/dark cycle (12 h light/12 h dark) and free access to food and water. The EE was realized by housing groups of eight female mice in larger cages (598 × 380 × 200 mm, *Type IV*) with more bedding and nesting material as described before (Nithianantharajah and Hannan, 2006; Hüttenrauch et al., 2016). Enriched cages were supplied with objects varying in color, size, shape, and texture. Physical activity was promoted by adding tunnels, climbing cubes, saucer wheels, and running wheels to the cages. Objects were rearranged three times a week over a period of 12 months in order to maintain novelty and complexity. For subsequent experiments, WT and TG female mice from different SE and EE cages were used in order to minimize cage and hormone cycle effects.

### Ethical approval

All procedures strictly adhered international standards for the care and use of laboratory animals and were approved by the local Animal Welfare and Ethics committee of the Country Commission Tübingen, Germany (TVA HG 4/11).

### Tissue preparation for immunohistochemistry and immunofluorescence staining

WT and TG female mice at the age of 8 and 12 months were deeply anesthetized with CO_2_ and intracardially perfused with phosphate-buffered saline (PBS, *pH* 7.4) and 4% paraformaldehyde (PFA, *pH* 7.4) prepared in PBS. Brains were removed and incubated in 4% PFA overnight at 4°C, transferred to 0.4% PFA the next day for a maximum period of one week, before embedding them in paraffin. Seven micrometer thick sagittal sections were prepared from paraffin-embedded brains and stored at room temperature until staining.

### Immunohistochemical staining

In order to detect human and murine alpha-synuclein, brain sections were deparaffinized in xylene and rehydrated using descending concentrations of ethanol. For demasking of antigens after fixation, slides were microwaved for 15 min in 10 mM citrate buffer (*pH* 6.0) then washed in PBS. Subsequently, endogenous peroxidase was inactivated by incubation in 0.3% H_2_O_2_ solution and unspecific binding was blocked using 5% normal serum for 45 min. Slides were incubated overnight with primary antibodies at 4°C (anti-human alpha-synuclein, 1:50, 15G7, Enzo Life Sciences), (anti-murine alpha-synuclein, 1:500, D37A6, Cell Signaling). The respective biotin-coupled secondary antibodies were added for 1 h. Sections were incubated with ABC reagent (ABC Kit, Vector) prepared at least 1 h before usage. For detection of target proteins, sections were washed (3 × 5 min in TBS) and incubated for 5 min with 3,3′-Diaminobenzidine (DAB) reagent (Sigma-Aldrich). Sections were washed for 10 min in *aq. dest*., dehydrated in a bath series of ascending ethanol concentrations, and finally placed in xylole before mounting with CV Mount (Leica Biosystems, Germany). Slides were dried for at least 24 h before microscopy using an *Axioplan 2* imaging microscope (Carl Zeiss, Germany).

### Immunofluorescence staining and imaging

After deparaffinization in xylene and descending concentrations of ethanol, antigen retrieval was performed as mentioned above and slides were washed in TBS. Blocking of unspecific binding was achieved using 10% of normal serum for 45 min at room temperature. Slides were incubated overnight with primary antibodies at 4°C (anti-human alpha-synuclein, 1:200, 15G7, Enzo Life Sciences), (anti-murine alpha-synuclein, D37A6, 1:250, Cell Signaling), (anti-MAP2, 1:100, SC-20172, Santa Cruz), and 1 h at room temperature with secondary antibodies coupled to a fluorescent dye. To visualize nuclei and somata, sections were counterstained with *NeuroTrace Blue* (1:200, Thermo Fisher Scientific) and *SYTOX* Green (1:30,000, Thermo Fisher Scientific) for 20 min, washed (3 × 5 min in TBS) and mounted with mowiol mounting medium (2.4 g Mowiol 4–88, 60 g Glycerol, 6 mL H_2_O, 12 mL 0.2 M Tris-Cl (*pH* 8.5) before mounting 2.5% DABCO added). Cover slips were stabilized with nail polish (*Quick dry top coat*, Cosnova, Germany) and slides dried at 4°C for at least 24 h before microscopy.

Fluorescent images were taken as *z*-stacks on a confocal laser-scanning microscope (*LSM 710*, Zeiss, Germany) using a 40x 1.4NA oil objective and the pinhole set to one airy unit. Images were taken with 12 bits depth, 1024 × 1024 pixels resolution, and a dwell time of 0.6 μs per pixel. Detector gain and laser power of each channel were adjusted appropriately, and a digital gain of one and no offset were used. *ZEN 2011* (Zeiss) imaging software was used.

### Tissue preparation for protein and RNA isolation

In order to assess protein and RNA levels, WT and TG mice were randomly chosen from different cages and sacrificed with cervical dislocation followed by head decapitation within 2 min from disturbing the home cage. Brain regions were immediately dissected on ice and snap frozen in liquid nitrogen.

### Western blot

RIPA buffer (radioimmunoprecipitation assay buffer, *pH* 7.4) supplemented with protease inhibitors (Complete protease inhibitor cocktail, Roche Applied Science) was used for protein extraction from hippocampal tissue (*n* = 3–6 animals per group) followed by measuring protein concentrations using Bradford assays (*Protein Assay Dye Reagent Concentrate*, Biorad, Germany). After protein separation on 12% Bis-Tris gels, nitrocellulose membranes were used for protein blotting for 1.5 h at 4°C. Subsequently, membranes were blocked with *Odyssey blocking solution* (LI-COR Biosciences) and incubated with primary antibodies at 4°C overnight (anti-human alpha-synuclein, 1:100, 15G7, Enzo Life Sciences), (anti-GFAP, 1:100, DAKO), (anti-NURR1, 1:250, sc-991, Santa Cruz), (anti-EGR1, 1:250, sc-189, Santa Cruz), (anti-synaptophysin, 1:2000, MAB368, Millipore), (anti-synapsin, 1:500, cs-2312, Cell Signaling), (anti-VAMP-1/2/3, 1:100, sc-133129, Santa Cruz), (anti-complexin 1/2, 1:250, sc-33603, Santa Cruz), (anti-PSD95, 1:1,000, ab12093, Abcam). Incubation with respective near-infrared fluorescent secondary antibody (IRDye 800CW or IRDye 680LT, *LI-COR* Bad Homburg, Germany) was followed by membrane detection and image quantification using *LI-COR* Odyssey imaging system and *Image Studio*, respectively.

### RNA sequencing

The polyadenylated fraction of RNA isolated from hippocampal tissue (*n* = 4 animals in each of the four experimental groups) was used for single-end RNA-seq. Total RNA and DNA were simultaneously extracted using the *AllPrep DNA/RNA Mini Kit* (Qiagen) using the manufacturer's protocol. Quality was assessed with an *Agilent 2100 Bioanalyzer*. Samples with high RNA integrity number (RIN > 8) were selected for library construction. Using the *TruSeq RNA Sample Prep Kit* (Illumina) and 500 ng of total RNA for each sequencing library, poly(A) selected single-end sequencing libraries (50 and 65 bp read length) were generated according to the manufacturer's instructions. All libraries were sequenced on an Illumina HiSeq 2500 platform at a depth of 10–20 million reads each. Library preparation and sequencing procedures were performed by the same individual, and a design aimed to minimize technical batch effects was chosen.

## Bioinformatics

### Quality control, alignment, and expression analysis

Read quality of RNA-seq data in fastq files was assessed using *FastQC* (v0.11.4) (Andrews, 2010) to identify sequencing cycles with low average quality, adaptor contamination, or repetitive sequences from PCR amplification. Reads were aligned using *STAR* (v2.4.2a) (Dobin et al., 2013) allowing gapped alignments to account for splicing against a custom-built genome composed of the *Ensembl Mus musculus* genome v82 and the human *SNCA* transgene. Alignment quality was analyzed using *samtools* (v1.1) (Li et al., 2009) and visually inspected in the *Integrative Genome Viewer* (v2.3.67) (Thorvaldsdóttir et al., 2013). Normalized read counts for all genes were obtained using *DESeq2* (v1.8.2) (Love et al., 2014). Transcripts covered with less than 50 reads were excluded from the analysis leaving 12,287 genes for determining differential expression in each of the pair-wise comparisons between experimental groups.

The 2 × 2 factorial design of the experiment was captured in a general linearized model in *DESeq2* modeling expression (t) as a function of genotype (g), the environment (e), and their interaction (g × e). Surrogate variable analysis (sva, v3.22.0) was used to minimize unwanted variation between samples (Leek et al., 2012). Given that differences in transcript abundances in brain tissue are often small in magnitude and *in vivo* RNA-seq data are deemed to be more variable (Maze et al., 2014), we set |log_2_ fold-change| ≥ 0.3 and adjusted *p*-value ≤ 0.15 to determine differentially expressed genes, as computationally predicted candidates down to the lower end of these thresholds could be confirmed in qPCR assays.

Gene-level abundances were derived from *DESeq2* as normalized read counts and used for calculating the log_2_-transformed expression changes underlying the expression heatmaps and the *k*-means clustering with ratios computed relative to the mean expression in WT_SE_. The *sizeFactor*-normalized counts provided by *DESeq2* also went into calculating nRPKMs (normalized Reads Per Kilobase per Million total reads) as a measure of relative gene expression as motivated before (Srinivasan et al., 2016). The *sizeFactors* further served in scaling estimated abundances derived from *Salmon* (v0.7.2) (Patro et al., 2016) when determining the transcript-level composition for individual genes. Transcript-level differential expression was further explored and verified with *kallisto* (v0.43.0) (Bray et al., 2016).

### Gene annotation, enrichment tests, and functional analyses

*DAVID* 6.7 and *WebGestalt 2017* were employed to identify overrepresented Gene Ontology terms and associated cellular functions in sets of differentially expressed genes (Huang da et al., 2009; Wang et al., 2013). Terms with a minimum number of three supporting candidates for smaller and ten for larger gene sets, their fold enrichment, and Benjamini-Hochberg adjusted *p*-value ≤ 0.1 are reported. The *SNCA* gene network was constructed in *GeneMANIA* (v3.4.1) (Warde-Farley et al., 2010) based on default parameters. Only first neighbors of *SNCA* in the network (without their interconnections) were selected. Grouping and coloring based on manually determined Gene Ontology categories of the genes. Transcription factor binding site (TFBS) analyses were carried out in *Pscan* (v1.4) (Zambelli et al., 2009) on the *Mus musculus* genome, considering −450 to +50 bp of promoter regions for motifs against the *JASPAR 2016* database. All Gene ID conversions were done using the *biomaRt* Bioconductor package (v2.30.0) querying v82 of the *Ensembl* database. Cell type-specific classifications were derived from the dataset provided through the Linnarsson lab (Zeisel et al., 2015). Only relevant hippocampal cell types were considered. The relative expression of a gene in the Linnarsson dataset was calculated as mean cell type-specific expression divided by total expression across all types. To estimate potential shifts in composition between neuronal and gilial cell types, the manually curated dataset from the Bonn group was utilized additionally (Halder et al., 2016).

### Reverse transcription-quantitative PCR (RT-qPCR)

RNA-sequencing results were validated using RT-qPCR with primers specific for *Nr4a2, Egr1, Arc, Wfs1, Tyro3, Gfap, Homer1a*, and *Homer1b/c* genes (see Supplementary Table 1 for primers sequences). One hundred nanogram of total RNA was used for the reverse transcription reaction (*QuantiTect Reverse Transcription* kit, Qiagen) following the manufacturer's instructions. The resulting cDNA was diluted (1:20) and 2 μl were then used for the qPCR assay, mixed with primers (0.5 μM) and *SYBR green* master mix (Qiagen). Relative expression was calculated based on the Pfaffl model (Pfaffl, 2001) after normalization to the geometric mean relative expression of two reference genes (*Tfrc* and *Sdha*), which were previously assessed for their stable expression using *BestKeeper* (Pfaffl et al., 2004), *Normfinder* (Andersen et al., 2004), and *Genorm* (Vandesompele et al., 2002). Data were calculated using Excel-based equations and further validated using *qBase* (Hellemans et al., 2007).

### Statistical analysis

Statistical comparisons for RT-qPCRs and protein assays were done in *GraphPad Prism* (v6.0). Two-way ANOVA was used to test for genotype, environment, and their interaction with Tukey's correction for multiple comparisons (see Supplementary Table 2 for ANOVA results). Differences between WT and TG mice at 6 and 12 months (protein analyses) were tested with two-way ANOVA followed by Bonferroni's correction. In protein assays with only two groups, unpaired two-tailed *t*-tests were applied using significance thresholds of *p* < 0.05. Animals were randomly allocated to experimental groups and chosen from different cages. No animals were excluded in any of the data reported.

## Results

### Overexpression of human *SNCA* disturbed the hippocampal transcriptome in transgenic mice

For this study, a BAC transgenic mouse model (TG) overexpressing the full-length human wildtype *SNCA* gene under its native promotor was used containing a construct described before (Yamakado et al., 2012; Nuber et al., 2013). In this transgenic model, human as well as murine alpha-synuclein protein were localized predominantly in the forebrain (Supplementary Figure 1A), with highest levels of human alpha-synuclein in the olfactory bulb, cortex, striatum, and hippocampus (Supplementary Figure 1B). Co-staining of hippocampal sections with either murine or human alpha-synuclein together with subcellular markers revealed similar subcellular distributions for murine and human alpha-synuclein in TG mice as well as for murine alpha-synuclein in WT and TG mice (Supplementary Figure 1C). Both forms of alpha-synuclein were mainly located in the neuropil. Other presynaptic proteins showed lower abundancies in the hippocampus of TG mice already at 6 months of age for synaptophysin (SYP) and synapsins (SYN), and at 12 months for synaptobrevins (VAMP-1/2/3) (Supplementary Figures 1D,E).

**Figure 1:**
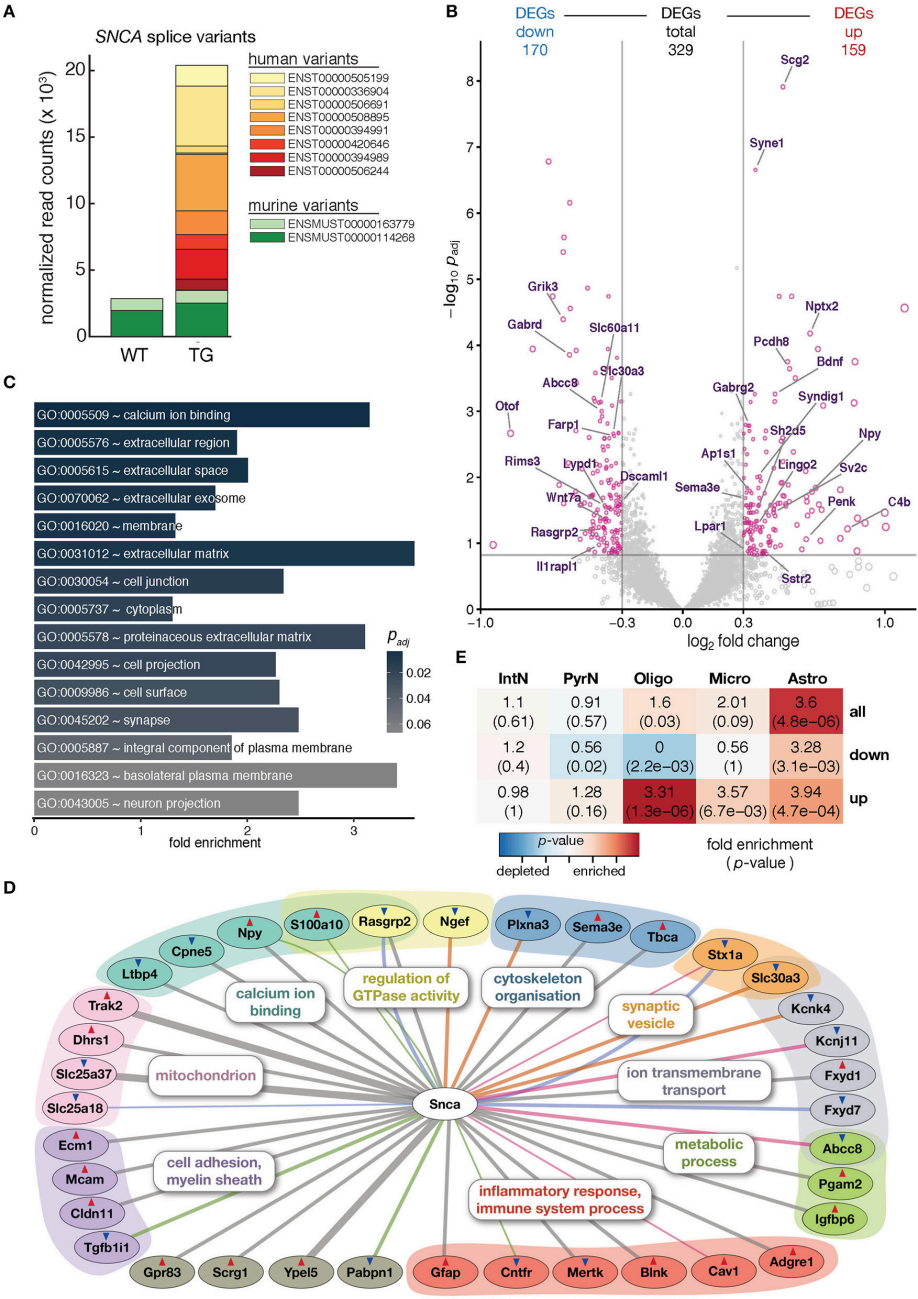
Overexpression of human *SNCA* altered the hippocampal transcriptome. **(A)** Composition and expression level of murine and human *SNCA* splice variants in hippocampus of 12-month-old WT and TG mice. **(B)** Volcano plot showing differences in gene expression between hippocampi of TG and WT mice. Pink dots highlight 329 genes differentially expressed above cut-offs of p_adj_ ≤ 0.15 and | log_2_ FC | ≥ 0.3 indicated by dashed lines. Genes with Gene Ontology-slim annotation *synapse* are labeled. Only murine genes plotted; for expression changes of endogenous and human *SNCA* see **(A)**. **(C)** Fold enrichment of overrepresented Gene Ontology terms among DEGs. **(D)** Known direct interactions of *SNCA* with 329 DEGs (*n* = 36). Type of interaction color-coded (gray, co-expression; pink, physical interaction; orange, MGI phenotype; green, PPI predicted; blue, co-localization). Genes arranged and colored based on Gene Ontology categories. Red and blue triangles indicate up- and downregulation of genes. **(E)** Cell type enrichment analysis of 329 DEGs indicating fold enrichment for genes attributed to interneurons (IntN), pyramidal neurons (PyrN), oligodendrocytes (Oligo), microglia (Micro), and astrocytes (Astro) (Zeisel et al., 2015). *p*-value represents significance in enrichment (red) or depletion (blue) over background by two-sided Fisher's exact test.

To assess molecular alterations of *SNCA* biology in TG animals, the hippocampal transcriptome of 12-month-old WT and TG animals (4 animals each) was profiled using deep-sequencing of poly A-enriched RNA and analyzed for differential gene expression. *SNCA* itself showed no changes in murine levels but an intriguing additional set of human *SNCA* splice variants that led to a more than six-fold overexpression in TG compared to WT animals (Figure 1A). Including *SNCA*, a total of 329 differentially expressed genes (DEGs, 159 up- and 170 downregulated) were identified (Figure 1B). Agreeing with the role of alpha-synuclein at presynapses (Lashuel et al., 2013), several genes annotated for the Gene Ontology-slim term *synapse* showed differential expression, some of them previously associated with Parkinson's disease including *Nptx2, Wnt7a*, and *Rims3* (Figure 1B) (Moran et al., 2008; Simunovic et al., 2009; Inestrosa and Arenas, 2010).

Among the 329 DEGs, *calcium ion binding*, in addition to *extracellular space, membrane*, and *synapse*, was the most significantly enriched Gene Ontology term (Figure 1C), in line with previous studies that describe alpha-synuclein overexpression to increase Ca^2+^ influx from the extracellular space, disrupt Ca^2+^ signaling, and affect its homeostasis (Danzer et al., 2007).

In addition, several other genes previously reported in the context of *SNCA* biology were among the DEGs when constructing the association graph based on co-expression, physical interaction, and additional characteristics (Figure 1D). Interestingly, the graph also contained DEGs attributed to glial cell types, including the astrocyte marker *Gfap* and the oligodendrocyte-specific *Cldn11* gene. To further explore dysregulation linked to individual cell types, DEGs were computationally related to cell type-specific expression data from single-cell RNA-seq experiments in mouse hippocampus (Zeisel et al., 2015). Intriguingly, upregulated DEGs were significantly enriched for genes attributed to oligodendrocytes, microglia, and astrocytes, respectively (Figure 1E). Comparing gene expression values for WT and TG samples against two cell type-specific data sets (Zeisel et al., 2015; Halder et al., 2016) gave a computational estimate of the cell type composition with no significant changes (Mann-Whitney *U*-test, two-tailed) between groups, suggesting a general homogeneity and no shift between neural and glial cells in the hippocampus of 12-month-old animals (Supplementary Figure 2).

These characteristics made the *SNCA* overexpressing mouse line an ideal model to assess molecular perturbations of *SNCA* biology at pre-symptomatic stages rather than capturing the state of a severely disturbed system as a consequence of neuronal loss. It further allowed investigating as to how exposure to an enriched environment induces its influence onto the regulatory framework along the gene-environment axis.

### Environmental enrichment induced neuronal genes including *Bdnf* in wildtype animals

**Figure 2:**
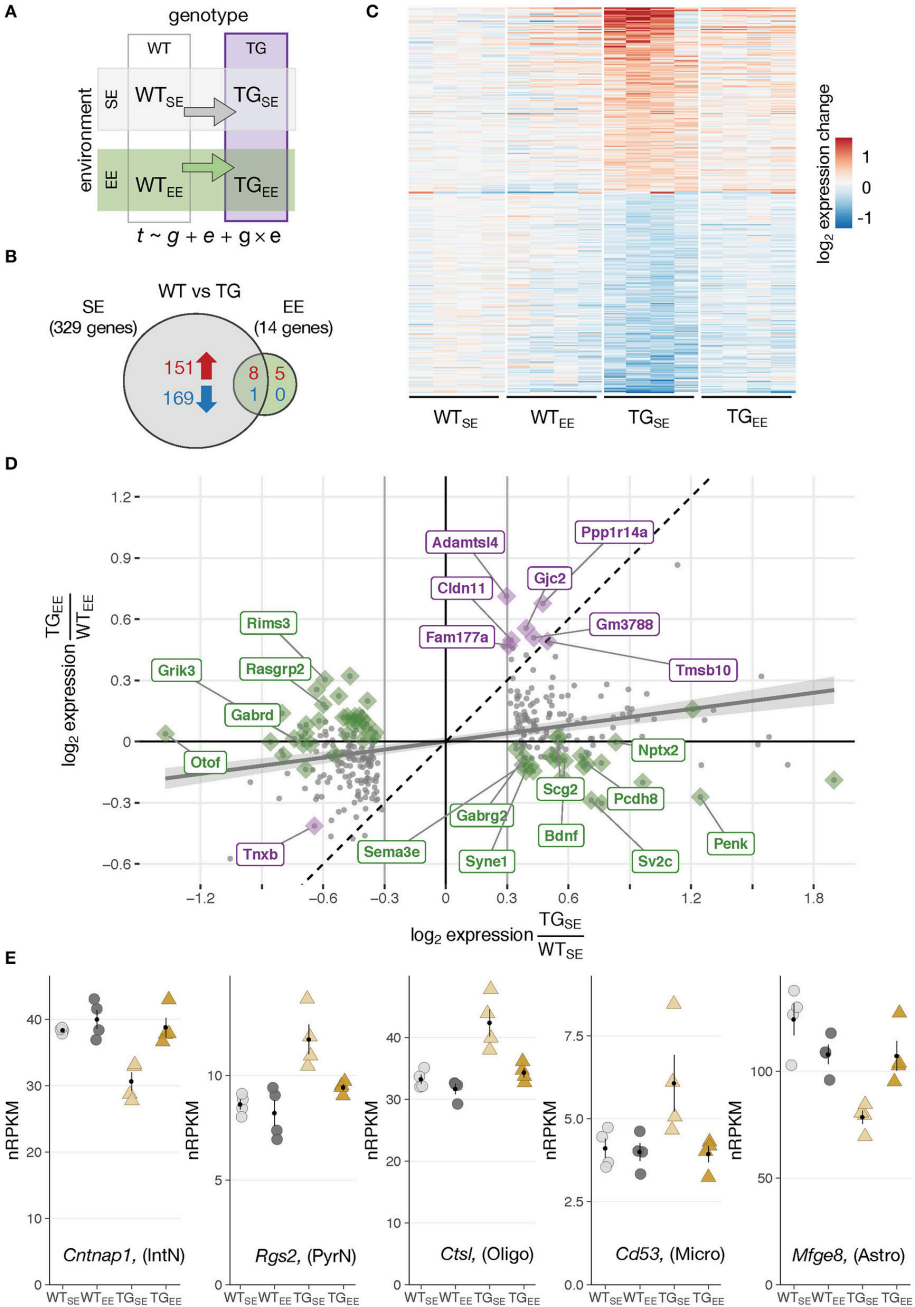
Environmental enrichment prevented disturbances in gene expression. **(A)** Schematic diagram of four experimental groups in a 2 × 2 factorial design with two genotypes (WT, TG) and two environmental conditions (SE, EE). WT_SE_, wildtype mice in standard environment; WT_EE_, wildtype mice in enriched environment; TG_SE_, transgenic mice in standard environment; TG_EE_, transgenic mice in enriched environment. Gene expression (t) was modeled as a function of genotype (g), environment (e), and their interaction (g × e). **(B)** Venn diagram comparing number of DEGs between TG_SE_/WT_SE_ and TG_EE_/WT_EE_. **(C)** Expression levels (log_2_ expression change relative to WT_SE_) across all experimental groups of 329 DEGs (TG_SE_/WT_SE_). Rows sorted by mean expression change in TG_SE_. **(D)** Scatter plot of log_2_ gene expression changes between WT and TG mice housed in standard (*x*-axis) and enriched environment (*y*-axis). Plotted in gray are 329 DEGs (TG_SE_/WT_SE_). Linear regression line with standard error shown in gray. Dashed line represents ordinary diagonal. Genes differentially expressed in both SE and EE labeled in purple. Dots highlighted in green indicate genes significantly affected by the environment based on the interaction term of the statistical model. **(E)** Expression levels in normalized reads per kilobase per million (nRPKMs) for selected genes plotted as individual data points with mean ± SEM. Assigned cell type specificity is indicated (Zeisel et al., 2015).

In order to study the impact of environmental enrichment in the context of *SNCA* overexpression, a 2 × 2 factorial design of experimental groups enabling cross-comparisons of genotype and environment was set up (Figure 2A, Supplementary Figure 3A). Specifically, the effects of a long-term EE and standard housing conditions (SE) were compared for WT and TG mice leading to four experimental animal groups (WT_SE_, WT_EE_, TG_SE_, TG_EE_). The EE, set up as a combination of increased physical activity, cognitive stimulation, and social interaction, was realized by housing larger cohorts of WT and TG animals directly after weaning for a period of 12 months in bigger cages with running wheels, tunnels, and various toy items that were rearranged three times a week (Supplementary Figure 3B).

In WT animals, the EE induced 41 DEGs (23 up- and 18 downregulated) (Supplementary Figures 4A,B) enriched for Gene Ontology terms like *morphogenesis involved in neuron differentiation, cognition*, and *learning and memory* (Supplementary Figure 4C) that have been implicated with EE effects before (van Praag et al., 2000). On a cell type-specific level, upregulated DEGs were significantly overrepresented among genes attributed to pyramidal neurons (Supplementary Figure 4D), agreeing with previous studies that describe enriched environments to impact on the morphology of this cell type (Gelfo et al., 2009). *Bdnf*, known for its promoting effect on cognitive function under environmental enrichment (Yuan et al., 2012), was upregulated after the long-term EE exposure (Supplementary Figures 4B,E). Intriguingly, the increased expression of *Bdnf* originated from the exon IV splice variant that has previously been described to be responsive to activity (Lauterborn et al., 1996). Although not significant according to the applied thresholds, genes investigated in the context of EEs before, including *Dlg4* (also known as *Psd95*), *Shank1*, and *Shank3* also showed trends of elevated expression in WT_EE_ animals (Supplementary Figure 4F).

### Environmental enrichment prevented transcriptional disturbances resulting from *SNCA* overexpression

Having explored effects of the overexpressed transgene and the EE individually, it was of particular interest to investigate these two factors in combination and shed light on the impact of environmental enrichment in transgenic animals. Intriguingly, when comparing TG to WT animals under EE conditions only 14 DEGs (13 up- and 1 downregulated) were detected in contrast to 329 DEGs identified under standard environmental conditions (Figures 2A,B). They were enriched for oligodendrocytic genes and overrepresented for the Gene Ontology terms *myelin sheath* as well as *cell junction*. Nine of the 14 DEGs overlapped with the 329 DEGs identified under SE conditions, indicating the environmental enrichment largely prevented transgene-induced perturbations in expression. Indeed, the heatmap of gene expression changes for the 329 DEGs across all experimental groups showed that expression levels in the TG_EE_ group remained near control levels (Figure 2C). Plotting gene expression ratios for all 329 DEGs in both environmental conditions put the nine overlapping genes close to the standard diagonal, indicating that their transgene-induced expression change was not affected by the EE (Figure 2D, highlighted in purple). In contrast, most other DEGs in the scatter plot were shifted towards the *x*-axis. This effect was most prominent for the set of genes identified through the interaction term of the statistical model, implying their differential expression in presence of *SNCA* overexpression was significantly influenced by the environment (Figure 2D, candidates highlighted in green). Among these genes, there were two primary response types to the EE: Firstly, genes that were prevented from expression changes and showed near control expression levels in TG_EE_ (Figure 2E; Supplementary Figure 5A). Secondly, there was a small subset of genes shifted toward the *x*-axis because they had a similar response to the transgene and the EE, leading to similar expression levels between TG_SE_, WT_EE_, and TG_EE_. Candidates of this response type included *Bdnf*, *Nptx2, Ptgs2* (also known as *Cox-2*), *Pcdh8* (also known as *Arcadlin*), and *C1ql2* (Supplementary Figure 5B), most of them known to be activity-regulated IEGs that mediate intercellular communication and signaling at the synapse (Loebrich and Nedivi, 2009).

### Environmental enrichment prevented transcriptional disturbances in glial cells

**Figure 3:**
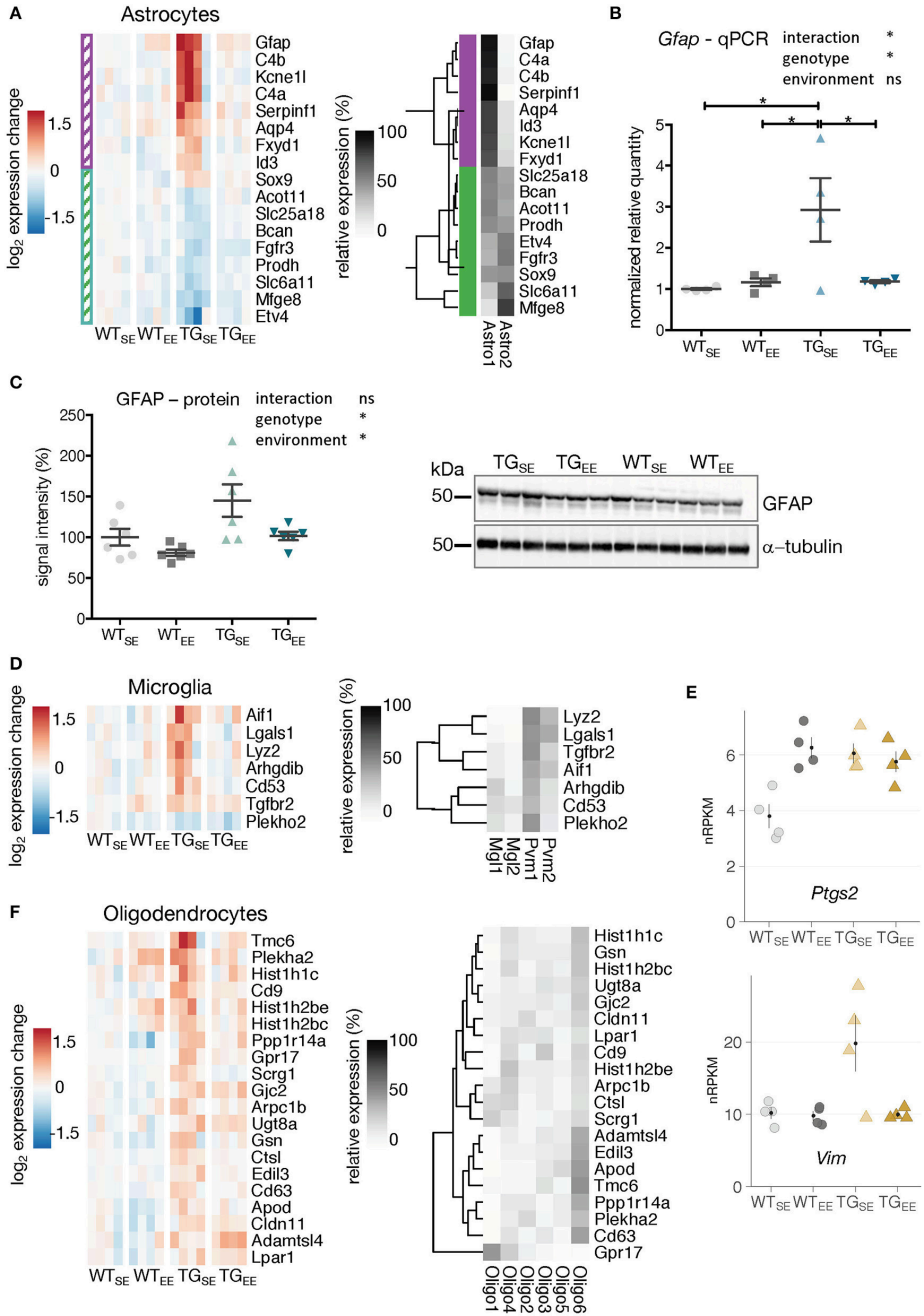
EE-induced prevention of transcriptional disturbances in glial cells. **(A)** Expression changes relative to WT_SE_ across experimental groups of astrocyte-associated DEGs (TG_SE_/WT_SE_) sorted by mean expression in TG_SE_. Same set of genes clustered by their relative read count in astrocytic subtypes (*astro1* and *astro2*) based on single-cell data (Zeisel et al., 2015). Gene assignment to either *astro1* or *astro2* color-coded in purple and green and indicated to the left of the gene expression heatmap. **(B)** Reverse transcription-quantitative PCR (RT-qPCR) data for *Gfap* shown as normalized quantities relative to WT_SE_ (*n* = 4 mice per group) and plotted as individual data points with mean ± SEM. Two-way ANOVA followed by Tukey's multiple comparisons test was performed *p < 0.05. **(C)** Representative protein blot of GFAP levels in hippocampal lysates across all experimental groups (*n* = 6 mice per group). Alpha-tubulin used for normalization. Graph shows quantification relative to WT_SE_ plotted as mean ± SEM. Two-way ANOVA was performed. **(D)** Expression changes relative to WT_SE_ across experimental groups of microglia-associated DEGs (TG_SE_/WT_SE_) sorted by mean expression in TG_SE_. Same set of genes clustered by their relative read counts in microglial subtypes based on single-cell RNA-seq data (Zeisel et al., 2015). **(E)** Expression values as nRPKMs for *Ptgs2* and *Vim* in each experimental group presented as individual data points with mean ± SEM. **(F)** Expression changes relative to WT_SE_ across experimental groups of oligodendrocyte-associated DEGs (TG_SE_/WT_SE_) sorted by mean expression in TG_SE_. Same set of genes clustered by their relative read counts in oligodendrocyte subtypes based on single-cell RNA-seq data (Zeisel et al., 2015).

To investigate the preventive effect of the EE on glial cells, DEGs attributed to astrocytes, microglia, and oligodendrocytes were analyzed for their expression in TG_EE_ animals. As shown in Figure 1E, up- as well as downregulated DEGs in TG_SE_ were significantly enriched for genes attributed to astrocytes. Upregulated genes included well-characterized astrocytic markers such as *Gfap* and *Aqp4* (Figure 3A). Downregulated DEGs included, among others, *Mfge8* (Figure 3A). *Gfap* and *Mfeg8* characterize two distinct subtypes of astrocytes, *astro1* and *astro2*, according to single-cell RNA-seq data (Zeisel et al., 2015). Intriguingly, all upregulated DEGs in TG_SE_ are strongly expressed in the *astro1* subtype, while all downregulated DEGs are expressed prominently in the *astro2* subtype (Figure 3A). For both up- and downregulated astrocytic DEGs, the enriched environment restored near control expression levels. This prevention was confirmed for *Gfap* using quantitative PCR (Figure 3B). In keeping, elevated GFAP protein levels were found in TG_SE_ animals and remained near control levels in TG_EE_ (Figure 3C).

Microglial DEGs that were mostly upregulated in TG_SE_ also showed near control expression levels in TG_EE_ (Figure 3D). The relative expression of microglia-attributed DEGs (Zeisel et al., 2015) across the sub-cell types (microglia: *Mgl1, Mgl2* and perivascular macrophages: *Pvm1, Pvm2*) showed their strongest expression levels to be in *Pvm1* (Figure 3D), which is in line with previous studies describing alterations in microglia as well as perivascular macrophages in Parkinson's disease patients (Wang et al., 2015). In addition, *Vim* and *Ptgs2* that are also associated with perivascular macrophages (Schiltz and Sawchenko, 2002; Mor-Vaknin et al., 2003) were upregulated (Figure 3E), consistent with reports of increased *Ptgs2* expression in Parkinson's disease patients and animal models (Teismann, 2012). While *Vim* showed near control expression levels in TG_EE_ animals, *Ptgs2* expression levels remained increased, reflecting its known activation by synaptic activity (Yamagata et al., 1993) as promoted by the EE and observed for other activity-depended genes, too (Supplementary Figure 5B).

In contrast to the effects in microglia and astrocytes, transcriptional disturbances in oligodendrocytes were only partially prevented by the EE (Figure 3F), consistent with oligodendrocytic genes being enriched among DEGs identified when comparing TG_EE_ with WT_EE_ (Figures 2B,D, highlighted in purple).

Taken together, the long-term environmental enrichment prevented changes in gene activity induced by *SNCA* overexpression in astrocytes and microglia, while the expression of genes attributed to oligodendrocytes remained largely altered.

### Sustained activation of immediate early genes in transgenic mice through environmental enrichment

**Figure 4:**
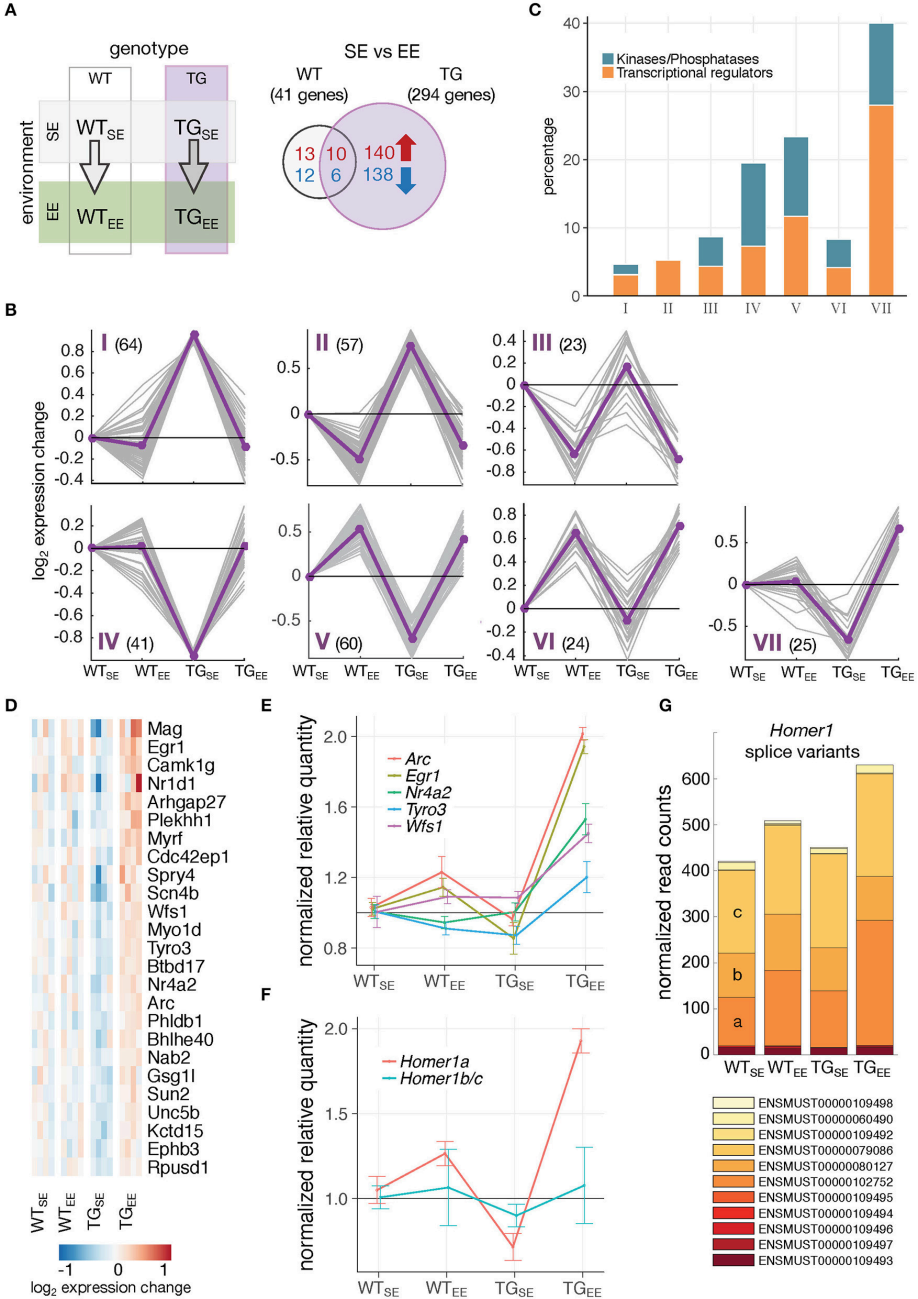
Distinct gene-environment response schemes. **(A)** Schematic diagram of four experimental groups comparing the effect of an enriched environment in wildtype and transgenic animals. Venn diagram comparing number of DEGs between WT_EE_/WT_SE_ and TG_EE_/TG_SE_. **(B)** Cluster analysis of the 294 DEGs from **(A)** using *k*-means on gene expression ratios relative to WT_SE_ of all experimental groups. Subplots show gene expression changes (log_2_ expression change) of each gene (gray lines) and cluster centroids (purple line). Number of DEGs in each cluster indicated in brackets. **(C)** Percentage of DEGs associated with the Gene Ontology terms *transcription regulator activity* and *protein kinase activity/phosphatases* for each cluster identified in **(B)**. **(D)** Cluster VII genes and their expression changes across all experimental groups relative to WT_SE_. **(E)** Validation of expression changes for *Egr1, Nr4a2*/*Nurr1, Arc, Tyro3*, and *Wfs1* using RT-qPCR. Normalized quantities relative to WT_SE_ (mean ± SEM) are plotted (*n* = 4 mice per group). For statistical details see Supplementary Table 2 and Supplementary Figure 6. **(F)** Validation of expression changes for splice variants *Homer1a* and *Homer1b/c* using RT-qPCR (*n* = 4 mice per group). Normalized quantities relative to WT_SE_ (mean ± SEM) are shown. For statistical details see Supplementary Table 2. **(G)** Composition and expression level of *Homer1* splice variants across experimental groups.

Next, it was of particular interest to reveal genes mediating the preventive effect. When comparing TG_EE_ with TG_SE_ animals, 294 DEGs (150 up- and 144 downregulated) were identified of which 16 were also differentially expressed in WT_EE_ animals (Figure 4A). To relate this response to the interplay of genotype and environment, expression profiles of all 294 DEGs were clustered across the four experimental groups, resulting in seven classes illustrating the main response types (Figure 4B). In cluster I, and mirror-imaged in cluster IV, genes showed disturbed expression in TG_SE_ that did not occur in TG_EE_. In contrast, genes in cluster III, and mirror-imaged in cluster VI, responded more generally to the EE, largely irrespective of the genotype and included the 16 genes found in the overlap in Figure 4A. Genes in clusters II and V exhibited both preventive and general EE-response characteristics. Cluster VII contained genes that were impaired in TG_SE_ and showed a sustained upregulation under EE in transgenic but not in wildtype animals. Intriguingly, 40 percent of the genes in cluster VII were associated with the Gene Ontology terms *transcriptional regulators* and *protein kinases/phosphatases* (Figure 4C), among them transcription factors like *Egr1* and *Nr4a2*/*Nurr1* as well as kinases such as *Tyro3* (Figure 4D).

Using quantitative PCR, we validated the distinct upregulation of several of these genes (Figure 4E; Supplementary Figure 6). Interestingly, nearly identical expression responses were observed for *Egr1* and two of its targets, *Nr4a2*/*Nurr1* and *Arc*, all of which are IEGs (Figure 4E). In addition to changes on gene level, the activity-dependent *Homer1a* splice variant also showed increased expression specifically in TG_EE_ animals, while the *Homer1b/c* isoforms remained near control levels (Figures 4F,G).

### Environmental enrichment largely restored reduced protein levels of EGR1 and NURR1 in transgenic animals

**Figure 5:**
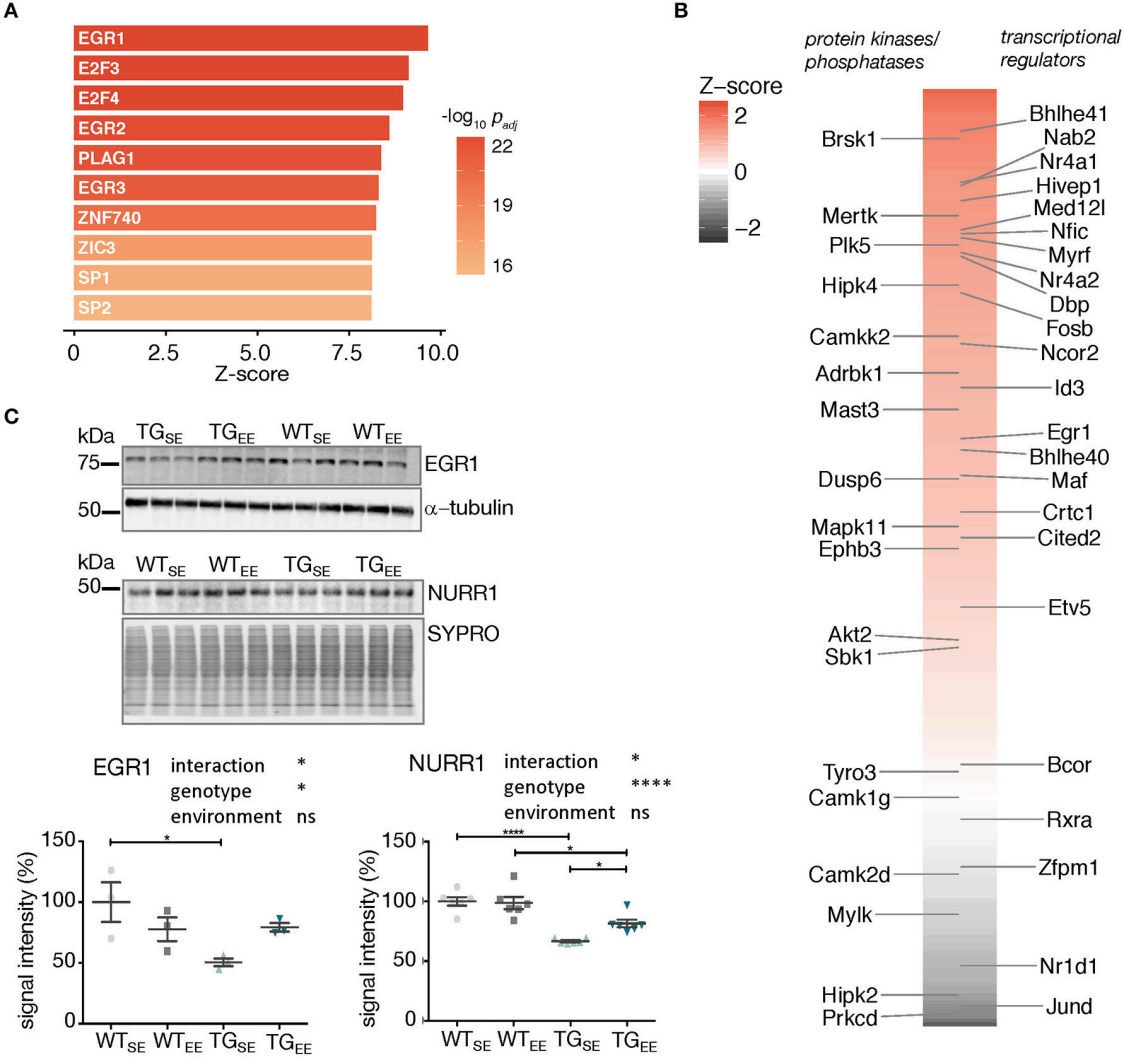
Associations between the regulatory framework around EGR1 and EE-induced prevention. **(A)** Promoter analysis of 294 DEGs (TG_EE_/TG_SE_) for overrepresented transcription factor binding sites. Depicted are *z*-scores for different position weight matrices of known transcription factors. Higher *z*-score reflect higher enrichment. Shown are the top ten significantly enriched matrices. **(B)** Color-coded and sorted *z*-scores of binding probabilities for EGR1 to the 294 DEGs (TG_EE_/TG_SE_). Labeled are DEGs associated with the Gene Ontology terms *transcription regulator activity* (Right) and *protein kinase activity/phosphatases* (Left). **(C)** Representative protein blot of EGR1 and NURR1 comparing protein levels in hippocampal lysates across experimental groups. alpha-tubulin and SYPRO protein blot stain used for normalization. Graph shows quantification (of *n* = 3 and *n* = 6 mice per group for EGR1 and NURR1, respectively) relative to WT_SE_ plotted as mean ± SEM. Two-way ANOVA followed by Tukey's multiple comparisons test was performed. *p < 0.05, ****p < 0.0001, ns = not significant. Note different loading order between blots.

To reveal potential transcriptional regulators involved in implementing the EE-induced prevention, the upstream promotor region of all 294 DEGs was scanned which revealed the EGR1 binding motif to be highly overrepresented (Figure 5A). Sorting the DEGs by their binding score for EGR1 put several other transcriptional regulators and kinases in the top ranks including targets such as *Nr4a2*/*Nurr1* (Figure 5B).

In order to test whether the sustained increase in expression of *Egr1* and *Nr4a2*/*Nurr1* observed in TG_EE_ animals was also reflected on protein level, hippocampal lysates of all experimental groups were compared in protein blotting. Interestingly, EGR1 and NURR1 showed reduced protein abundances in TG_SE_ animals that were largely restored through the EE (Figure 5C). This significant interaction between genotype and environment (Supplementary Table 2) suggests their EE-induced increase in gene expression was able to compensate for impaired protein levels and possibly activated down-stream processes that contributed to the observed prevention of disturbances caused by *SNCA* overexpression.

To explore whether the enriched environment prevented transcriptional disturbances by also directly influencing the alpha-synuclein load, we examined its RNA and protein levels along with other synaptic markers. However, both murine and human *SNCA* splice variants as well as human alpha-synuclein protein, and other tested presynaptic markers remained virtually unchanged under EE exposure (Supplementary Figure 7). This supports the hypothesis the EE-induced prevention was realized not directly through reduction of *SNCA* levels but rather by downstream cellular processes that were activated through the regulatory framework around *Egr1* and *Nr4a2*/*Nurr1* that mitigated disturbances despite a persistent alpha-synuclein load.

## Discussion

**Figure 6:**
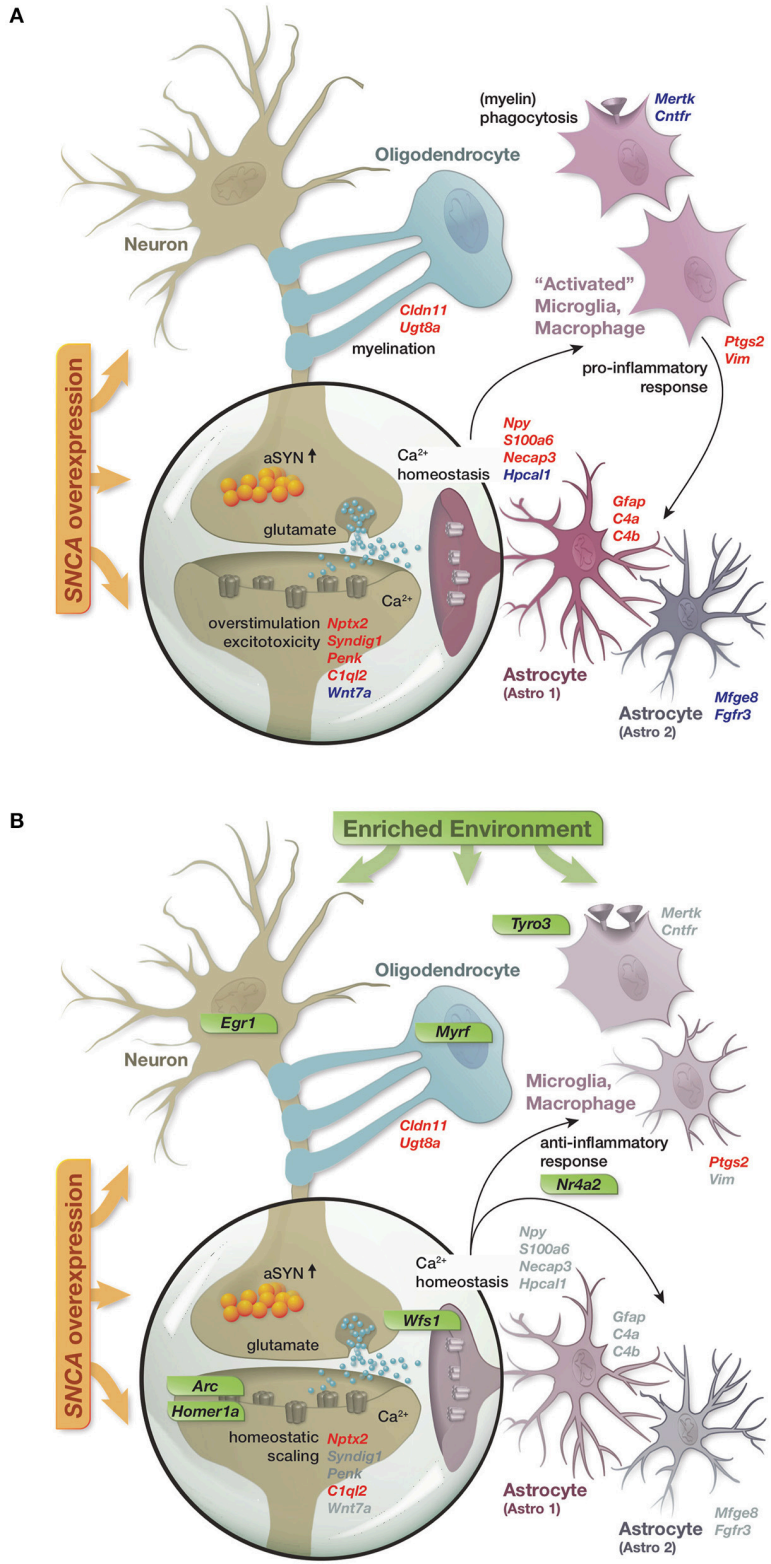
Graphical summary. **(A)** Schematic of perturbations in gene activity caused by *SNCA* overexpression and their attribution to distinct hippocampal cell types. Up- and down-regulation encoded as red and blue gene labels. **(B)** Prevention of *SNCA*-induced perturbations in gene activity through the long-term enriched environment. Gray gene labels indicate close to control expression levels. Genes specifically activated in response to the EE in the context of *SNCA* overexpression labeled in green.

In summary, the reported results lead us to propose a model in which *SNCA* overexpression disturbed the expression of a diverse set of genes, many of them linked to cellular functions previously described in the context of alpha-synuclein biology and attributed to distinct hippocampal cell types (Figure 6A). These disturbances in gene activity were accompanied by reduced levels of several presynaptic proteins and the immediate early genes EGR1 and NURR1 as well as an increase in the astrocytic marker GFAP, collectively hinting at impairments of synaptic function, activity-dependent signaling, and activation of astrocytes. Long-term exposure to an enriched environment largely prevented these disturbances in gene activity and restored protein levels of EGR1, NURR1, and GFAP despite a persistent alpha-synuclein load. Additionally, a group of genes responded to the enriched environment specifically in transgenic animals and potentially mediated the preventive effect. Among these genes were transcription factors such as *Egr1* and *Nr4a2*/*Nurr1*, capable to regulate larger sets of genes, as well as specific modulators including *Arc, Homer1a, Wfs1*, and *Tyro3* (Figure 6B).

Specifically, *Egr1* as a zinc finger transcription factor and an IEG is involved in maintaining long-term potentiation in the hippocampus and in consolidating several forms of memory (Knapska and Kaczmarek, 2004). IEGs show increased expression upon synaptic activity of glutamate receptors and elevated intracellular Ca^2+^ levels (Minatohara et al., 2015) and have been found upregulated through enriched environments (Pinaud, 2004). Intriguingly, we found *Egr1* and other IEGs responding specifically in TG_EE_ mice, agreeing with reports of similar effects in a mouse model for Alzheimer's disease (Lazarov et al., 2005). The common response scheme in both models could result from an impairment on RNA and/or protein level of *Egr1* and other IEGs in the disease context under standard conditions. Indeed, lower *Egr1* mRNA and protein levels in our mouse model are in line with downregulated RNA levels of *Egr1* in hippocampal tissue of Alzheimer's disease models (Dickey et al., 2003). However, it remains to be elucidated whether the reduction of EGR1 was a direct effect of *SNCA* overexpression or a secondary consequence of synaptic dysfunction (Schirinzi et al., 2016), which could in part be reflected by reduced presynaptic protein levels that we observed. IEGs being implicated and impaired in this context seems plausible given their role in synaptic plasticity, activity-induced signaling, calcium homeostasis, and gene expression that are known to be affected in the unfolding of synaptic dysfunction (Greer and Greenberg, 2008). Since these cellular processes are also responsive to activity-related stimulation from, for example, enriched environments, the upregulation of IEGs like *Egr1*, its nearly restored protein level, and the observed activation of its downstream targets could represent a mechanism how beneficial influences of the EE were integrated into the regulatory program and prevented perturbations from *SNCA* overexpression.

As part of this IEG regulatory framework, EGR1 target genes are linked to synaptic vesicle transport, clathrin-mediated endocytosis, transmission of signals elicited by Ca^2+^ influx, and synaptic plasticity (Koldamova et al., 2014). Among these targets, *Arc* and *Homer1a*, both IEGs too, function in synaptic scaling and removal of AMPA receptors from the plasma membrane (Hu et al., 2010; Korb and Finkbeiner, 2011). Their increased expression in TG_EE_ mice suggests rescaling efforts of pyramidal neurons to compensate for synaptic alterations including reduced presynaptic protein levels and perturbed expression of genes such as *Nptx2, Syndig1, Penk, Wnt7a*, and *C1ql2* involved in glutamatergic synaptic transmission.

Directly linked to synaptic transmission and glutamate signaling are intracellular Ca^2+^ levels (Rao and Finkbeiner, 2007) that are altered in the context of Parkinson's disease (Schapira, 2013). Here, we found *calcium ion binding* to be the most overrepresented Gene Ontology term among DEGs in TG_SE_ mice. In contrast, calcium-associated genes remained largely undisturbed in TG_EE_ animals, suggesting properly maintained Ca^2+^ levels, which was accompanied by upregulation of *Wfs1* and its capacity to increase calcium reuptake in the ER (Takei et al., 2006).

Imbalanced Ca^2+^ levels and aberrant synaptic signaling can impact neighboring astrocytes (Bazargani and Attwell, 2016). Using cell type-specific classification data (Zeisel et al., 2015), we identified specific enrichments of DEGs in TG_SE_ mice that are attributed to microglia, astrocytes, and oligodendrocytes, respectively. In fact, we linked differentially expressed genes to individual sub-cell types and found upregulated astrocytic genes including *Gfap* to be associated with the *astro1* subtype, and downregulated astrocytic genes including *Mfge8* to be attributed to the *astro2* subtype (Zeisel et al., 2015). This finding agrees with recent reports on distinct sub-cell types of neurotoxically reactive astrocytes that are activated in the context of neurodegeneration (Liddelow et al., 2017). Our data, obtained in a pre-symptomatic model with no detectable alpha-synuclein aggregates and no indication of a shifted cell type composition due to neuronal loss further strengthens the emerging concept from an increasing number of reports that glial cell activation is a contributing factor in early stages of disease unfolding rather than a consequence of aggregate formation in later stages of the pathogenesis (Halliday and Stevens, 2011), an idea also put forward for Alzheimer's disease (Hong et al., 2016).

Most of the disturbances in gene activity attributed to microglia and astrocytes were prevented in TG_EE_ mice. This was accompanied by increased expression of *Nr4a2*/*Nurr1* in TG_EE_ animals and its suggested anti-inflammatory capacity (Saijo et al., 2009). Similar to the effect observed for EGR1, protein levels of NURR1 were reduced in TG_SE_ mice but largely restored under environmental enrichment. Consistently, lower protein levels of NURR1 have also been identified in Parkinson's disease patients (Chu et al., 2002), and functional impairments of the gene have been implicated in the pathology of Parkinson's disease (Jiang et al., 2005). Further, reduced *Nr4a2*/*Nurr1* expression has been associated with inflammatory processes in the context of *SNCA* overexpression (Saijo et al., 2009), and its elevated expression with protecting dopaminergic neurons in the midbrain (Decressac et al., 2013). Here, we suggest these effects of *Nr4a2*/*Nurr1* are also applicable to the hippocampus.

Taken together, several studies by other groups have highlighted the neuroprotective effects of EE in different toxin-induced models of Parkinson's disease (Bezard et al., 2003; Faherty et al., 2005; Jadavji et al., 2006; Steiner et al., 2006; Anastasia et al., 2009; Goldberg et al., 2011; Klaissle et al., 2012; Jungling et al., 2017). Besides improved motor performance, more surviving dopaminergic neurons, increased striatal expression of *Gdnf* and *Bdnf*, and decreased levels of dopamine transporter were found in enriched animals, but the underlying mechanism on a gene regulatory level are largely unknown. Here, our results in a transgenic *SNCA* overexpressing model indicate that exposure to a long-term enriched environment is capable of entertaining an increased expression of a small set of genes, several of which are direct targets of or converge to the immediate early gene regulatory framework. Their concerted activation compensates for perturbations in the unfolding of synucleinopathies and restores a largely normalized expression profile. From this regulatory network, several genes emerge as pivotal and represent interesting targets to investigate their therapeutic potential. Utilizing their compensatory capacity by mimicking beneficial cues of enriched environments before such gene-environment mechanisms are capped or break as a consequence of neuronal loss could open novel avenues for treating synucleopathies and related disorders early on.

## Availability of data and materials

RNA-seq data files have been uploaded to GEO database and are available under the accession number GSE96961.

## Authors contributions

JS-H initiated the study and designed the experiments with OR and NC. ZW, SS, NC, and CR performed the experiments. TH and JS-H were responsible for computational analyses of the data. IE and PK guided imaging as well as molecular characterizations of the mouse model. ZW, TH, and JS-H wrote the paper. All authors read and approved the manuscript.

### Conflict of interest statement

The authors declare that the research was conducted in the absence of any commercial or financial relationships that could be construed as a potential conflict of interest.

## Supplementary material

Supplementary Figure 1 Characteristics of the transgenic mouse model overexpressing human *SNCA*. **(A)** Immunostaining for alpha-synuclein on 7 μm thick sagittal mouse brain sections embedded in paraffin using an antibody specific for murine and human alpha-synuclein protein in 8-month-old WT and TG animals. **(B)** Levels of human alpha-synuclein in different brain regions compared using protein blots. GAPDH used as loading control. Ob, olfactory bulb; Cb, cerebellum; Sc, spinal cord; Bs, brain stem; Mb, midbrain; St, striatum; Hi, hippocampus; Cx, cortex. **(C)** Seven micrometer thick sagittal sections from paraffin-embedded mice brains immunostained for murine alpha-synuclein (red), human alpha-synuclein (red), and MAP2 (green) in 12-month-old WT and TG animal. Nuclei (white) and somata (blue) counterstained with SYTOX nucleic acid stain and Neurotrace Nissl stain, respectively. A no primary antibody control (neg.) shown on the right of each panel. Images of the CA1 area in hippocampus acquired with a confocal laser-scanning microscope at 40x magnification. **(D)** Representative protein blots of SYP, SYN and VAMP-1/2/3 levels detected in hippocampal lysates of 6- and 12-month-old mice (*n* = 3–6 mice per group). alpha-tubulin was used for normalization. **(E)** Graph shows quantification of protein blots in **(D)** relative to WT_12m_. Two-way ANOVA followed by Bonferroni's multiple comparisons test was performed. Data presented as mean ± SEM. ^*^*p* < 0.05, ^***^*p* < 0.001. SYN, synaptophysin; Pan-SYN, pan-synapsin; VAMP1/2/3, synaptobrevins.

Supplementary Figure 2 Homogeneous cell type composition across samples. **(A)** Cell type-specific gene expression distributions for WT_SE_ and TG_SE_ samples. Boxplots show geometric mean as well as 10th, 25th, 75th, and 90th quantile of nRPKM values for all genes classified based on single-cell data (Zeisel et al., 2015). Number of genes per cell type in brackets. **(B)** Cell type-specific gene expression distributions for WT_SE_ and TG_SE_ samples. Boxplots show geometric mean as well as 10th, 25th, 75th, and 90th quantile of nRPKM values for a manually curated gene set classified as neuronal or glial (Halder et al., 2016). Number of genes per cell type in brackets.

Supplementary Figure 3 Experimental study design and enriched environment setup. **(A)** Experimental timeline. After birth, WT and TG mice were kept in SE for three weeks during weaning, then housed in either the standard or enriched environment to 12 months of age. **(B)** Pictures of the EE cages. Left shows representative layout of an EE cage with toys and running wheels that were rearranged three times a week. Right highlights size differences of cages. Larger rat cages (*Typ IV*, 598 × 380 × 200 mm) were used to house eight female mice/cage in the EE, standard mice cages (*Typ II long*, 365 × 207 × 140 mm) were used to house four female mice/cage in the SE.

Supplementary Figure 4 Environmental enrichment modulated neuronal pathways and enhanced expression of *Bdnf* in WT mice. **(A)** Number of differentially expressed genes between WT_EE_ and WT_SE_. **(B)** Expression levels (log_2_ of expression change relative to WT_SE_) per animal of 41 genes DEGs hierarchically clustered. **(C)** Fold enrichment of overrepresented Gene Ontology terms among DEGs. **(D)** Cell type enrichment analysis of DEGs indicating fold enrichment for genes attributed to interneurons (IntN), pyramidal neurons (PyrN), oligodendrocytes (Oligo), microglia (Micro), and astrocytes (Astro). *p*-value represents significance in enrichment (red) or depletion (blue) over background by two-sided Fisher's exact test. **(E)** Splice variant composition and expression level of *Bdnf*. **(F)** Expression levels of selected synaptic markers shown as individual data points with mean ± SEM.

Supplementary Figure 5 Two primary response types to the environmental enrichment in the context of *SNCA*-induced alterations. **(A)** Expression levels of selected genes that were prevented from expression changes and showed near control expression levels in TG_EE_ plotted as individual data point with mean ± SEM. **(B)** Expression levels of selected genes with a similar response to the transgene and the EE plotted as individual data point with mean ± SEM.

Supplementary Figure 6 Validation of RNA-seq results using RT-qPCR. RNA sequencing results of selected targets identified in Cluster VI and VII (Figure 4) were verified by RT-qPCR. **(Left)** shows RT-qPCR normalized quantities relative to WT_SE_ (individual data points with mean ± SEM). **(Right)** Shows expression levels in normalized reads per kilobase per million (nRPKMs) as individual data points with mean ± SEM based on RNA-seq data. For RT-qPCR data, two-way ANOVA with Tukey's *post hoc* test was performed.

Supplementary Figure 7 Alpha-synuclein load was unaffected by environmental enrichment. **(A)** Composition and expression level of murine and human *SNCA* splice variants across experimental groups. **(B)** Representative protein blot of human alpha-synuclein protein levels in hippocampal lysates of 12-month-old TG_SE_ and TG_EE_ mice. *SYPRO* protein blot stain used for normalization (*n* = 3, unpaired *t*-test, ns = not significant). **(C)** Representative protein blots of pre- and post-synaptic protein levels detected in hippocampal lysates across experimental groups (*n* = 3–6 mice per group). alpha-tubulin was used for normalization. Graph shows quantification relative to WT_SE_ plotted as mean ± SEM. Two-way ANOVA followed by Tukey's multiple comparisons test was performed. ^*^*p* < 0.05, ^**^*p* < 0.01, ^***^*p* < 0.001, ns = not significant. Note different loading order between blots. See Supplementary Table 2 for details. CS, citrate synthase; SYP, synaptophysin; Pan-SYN, pan-synapsin; CPLX, complexin.

Supplementary Table 1 Primer list. Primers used for RT-qPCR validation of RNA-sequencing, along with genotyping primers used in PCR and qPCR assays.

Supplementary Table 2 Comprehensive two-way ANOVA test results. Two-way ANOVA results for protein and RT-qPCR assays.

## Abbreviations

DEG: differentially expressed gene
EE: enriched environment
IEG: immediate-early gene
SE: standard environment
TG: transgenic
WT: wildtype.
