# Supplementary figures and images for: Environmental Enrichment Prevents Transcriptional Disturbances Induced by Alpha-Synuclein Overexpression
Source: Front Cell Neurosci. 2018 Apr 24;12:112. doi: 10.3389/fncel.2018.00112 (PMC5932345; doi:10.3389/fncel.2018.00112)

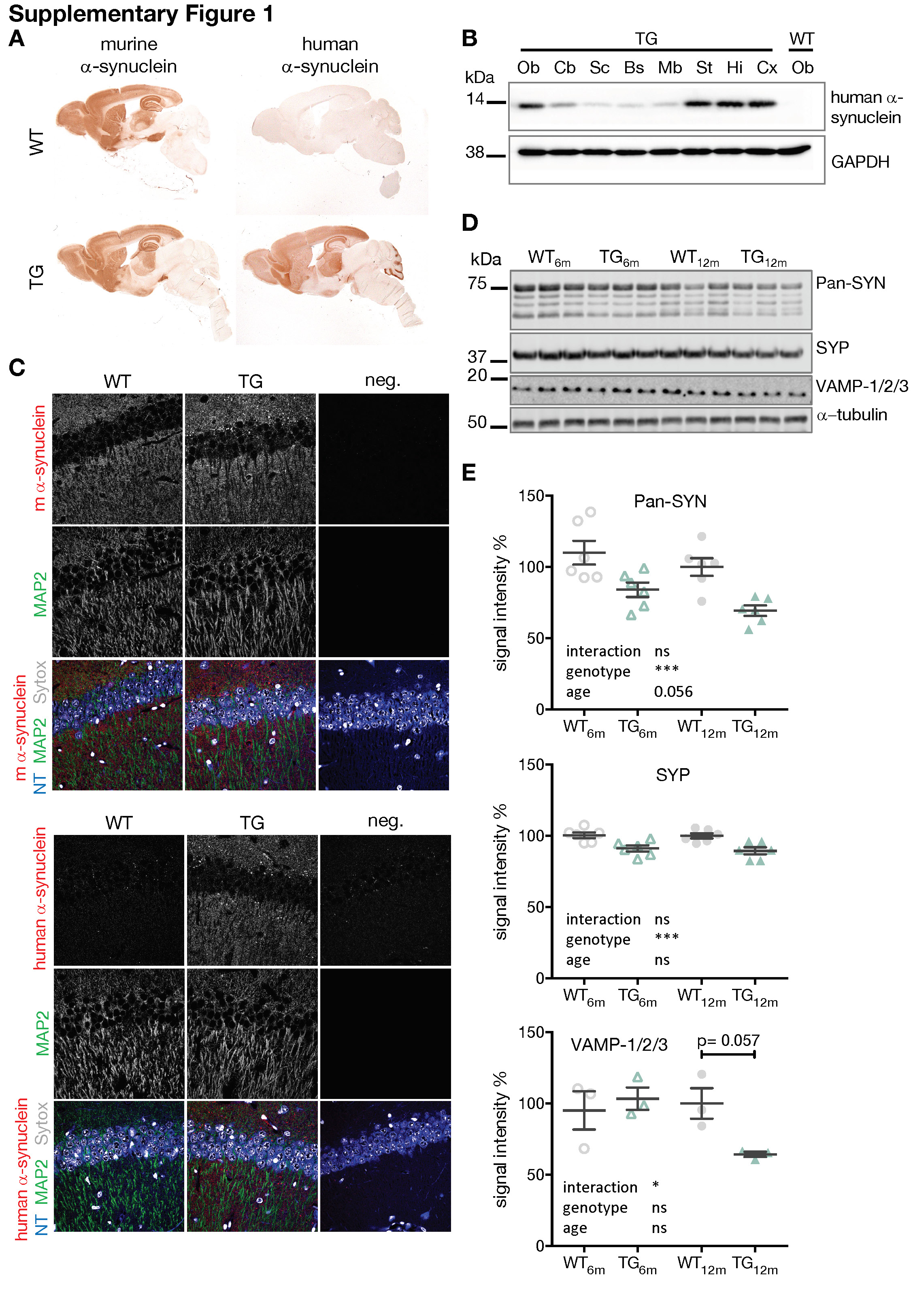

Supplement: Supplementary Figure 1 — Characteristics of the transgenic mouse model overexpressing human SNCA. (A) Immunostaining for alpha-synuclein on 7 μm thick sagittal mouse brain sections embedded in paraffin using an antibody specific for murine and human alpha-synuclein protein in 8-month-old WT and TG animals. (B) Levels of human alpha-synuclein in different brain regions compared using protein blots. GAPDH used as loading control. Ob, olfactory bulb; Cb, cerebellum; Sc, spinal cord; Bs, brain stem; Mb, midbrain; St, striatum; Hi, hippocampus; Cx, cortex. (C) Seven micrometer thick sagittal sections from paraffin-embedded mice brains immunostained for murine alpha-synuclein (red), human alpha-synuclein (red), and MAP2 (green) in 12-month-old WT and TG animal. Nuclei (white) and somata (blue) counterstained with SYTOX nucleic acid stain and Neurotrace Nissl stain, respectively. A no primary antibody control (neg.) shown on the right of each panel. Images of the CA1 area in hippocampus acquired with a confocal laser-scanning microscope at 40x magnification. (D) Representative protein blots of SYP, SYN and VAMP-1/2/3 levels detected in hippocampal lysates of 6- and 12-month-old mice (n = 3–6 mice per group). alpha-tubulin was used for normalization. (E) Graph shows quantification of protein blots in (D) relative to WT12m. Two-way ANOVA followed by Bonferroni's multiple comparisons test was performed. Data presented as mean ± SEM. *p < 0.05, ***p < 0.001. SYN, synaptophysin; Pan-SYN, pan-synapsin; VAMP1/2/3, synaptobrevins. [file Image_1.jpeg]

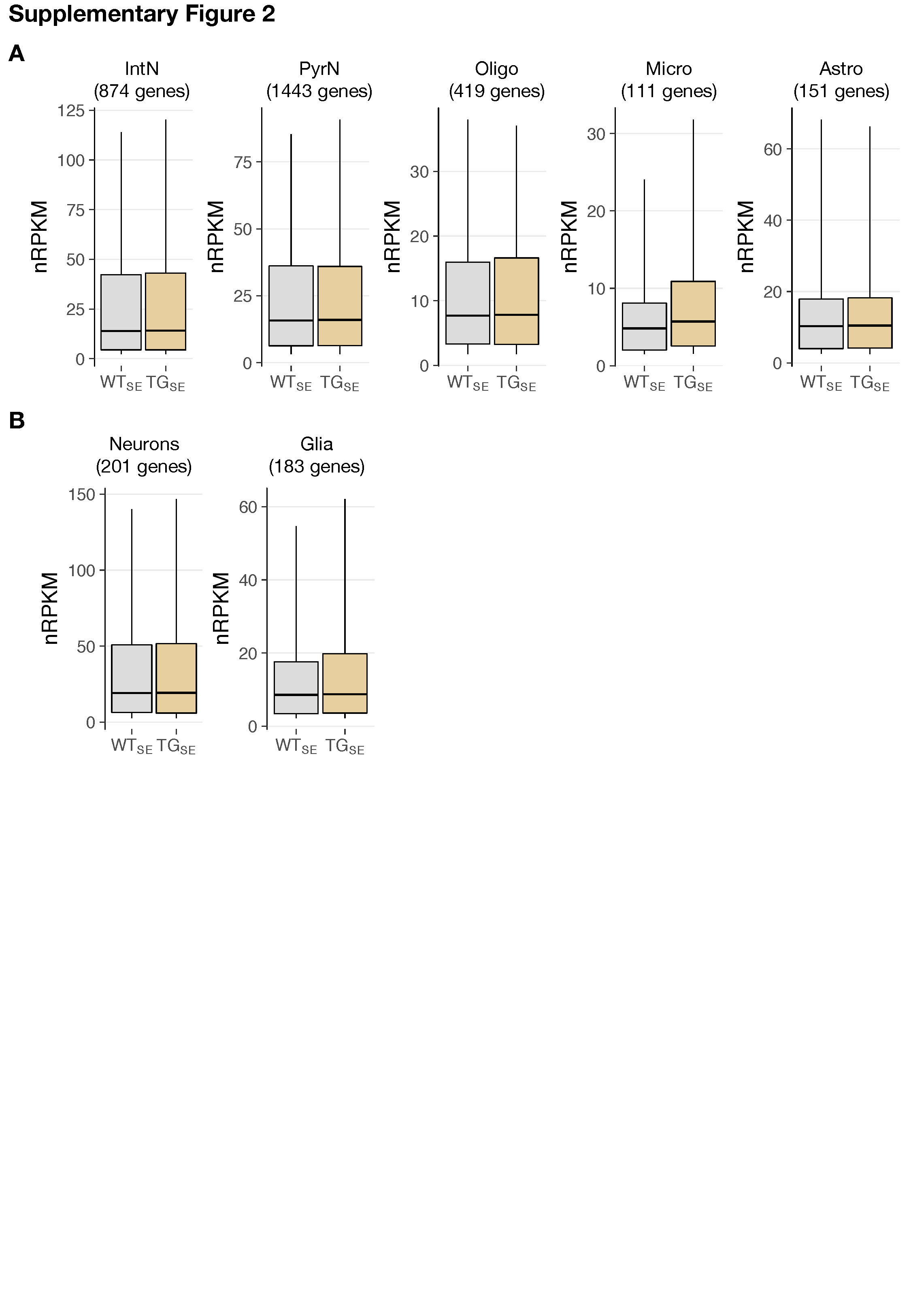

Supplement: Supplementary Figure 2 — Homogeneous cell type composition across samples. (A) Cell type-specific gene expression distributions for WTSE and TGSE samples. Boxplots show geometric mean as well as 10th, 25th, 75th, and 90th quantile of nRPKM values for all genes classified based on single-cell data (Zeisel et al., 2015). Number of genes per cell type in brackets. (B) Cell type-specific gene expression distributions for WTSE and TGSE samples. Boxplots show geometric mean as well as 10th, 25th, 75th, and 90th quantile of nRPKM values for a manually curated gene set classified as neuronal or glial (Halder et al., 2016). Number of genes per cell type in brackets. [file Image_2.jpeg]

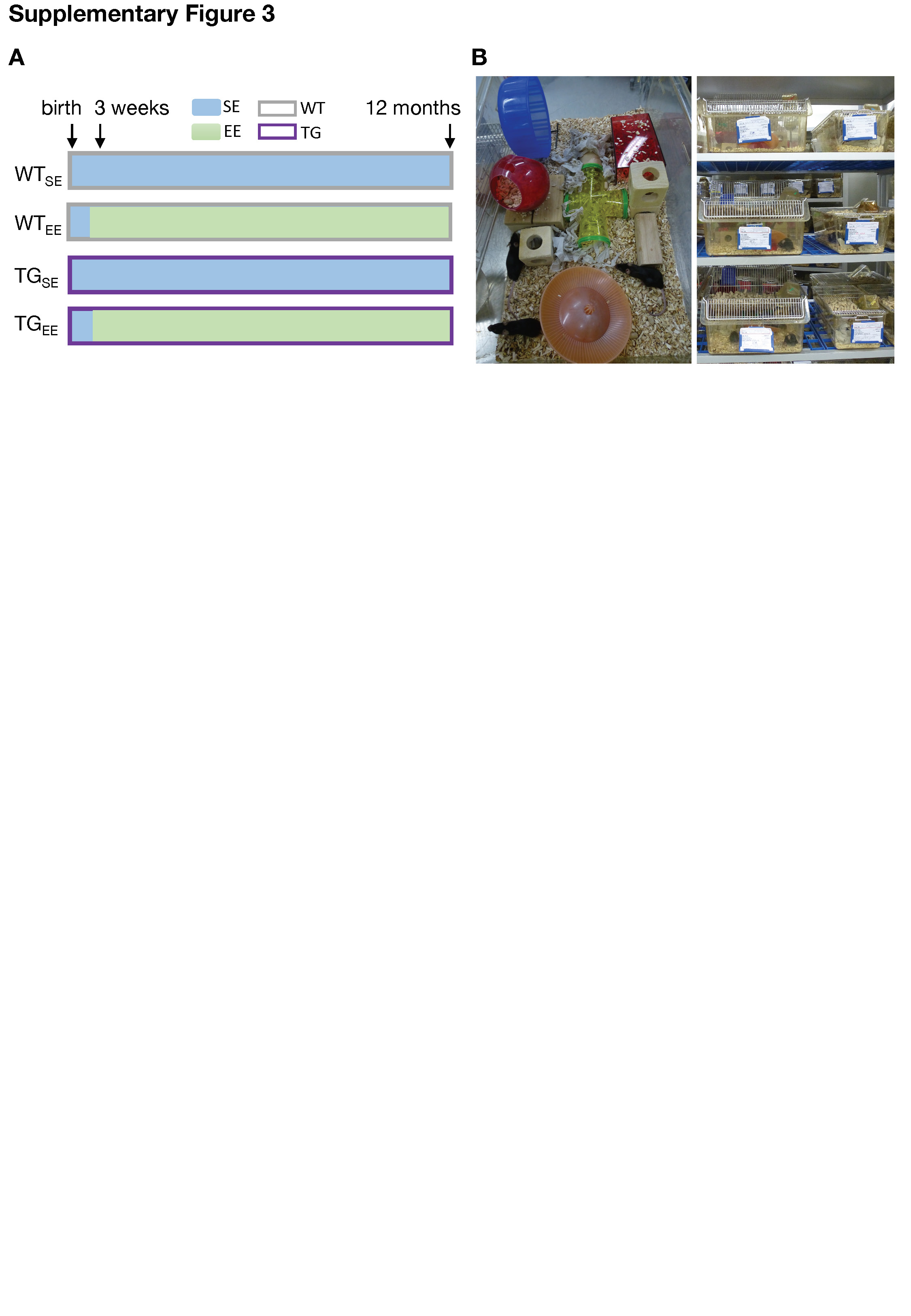

Supplement: Supplementary Figure 3 — Experimental study design and enriched environment setup. (A) Experimental timeline. After birth, WT and TG mice were kept in SE for three weeks during weaning, then housed in either the standard or enriched environment to 12 months of age. (B) Pictures of the EE cages. Left shows representative layout of an EE cage with toys and running wheels that were rearranged three times a week. Right highlights size differences of cages. Larger rat cages (Typ IV, 598 × 380 × 200 mm) were used to house eight female mice/cage in the EE, standard mice cages (Typ II long, 365 × 207 × 140 mm) were used to house four female mice/cage in the SE. [file Image_3.jpeg]

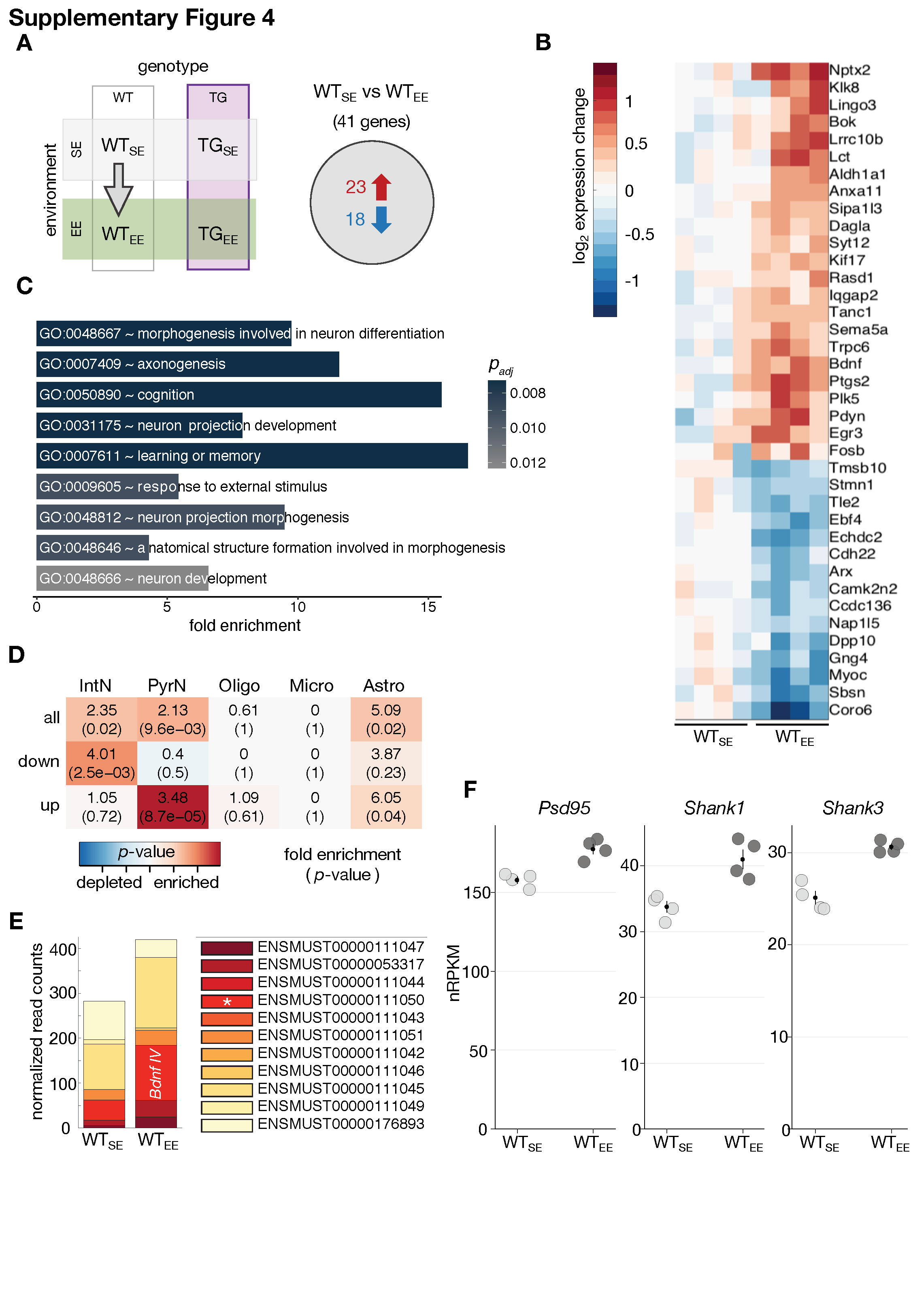

Supplement: Supplementary Figure 4 — Environmental enrichment modulated neuronal pathways and enhanced expression of Bdnf in WT mice. (A) Number of differentially expressed genes between WTEE and WTSE. (B) Expression levels (log2 of expression change relative to WTSE) per animal of 41 genes DEGs hierarchically clustered. (C) Fold enrichment of overrepresented Gene Ontology terms among DEGs. (D) Cell type enrichment analysis of DEGs indicating fold enrichment for genes attributed to interneurons (IntN), pyramidal neurons (PyrN), oligodendrocytes (Oligo), microglia (Micro), and astrocytes (Astro). p-value represents significance in enrichment (red) or depletion (blue) over background by two-sided Fisher's exact test. (E) Splice variant composition and expression level of Bdnf. (F) Expression levels of selected synaptic markers shown as individual data points with mean ± SEM. [file Image_4.jpeg]

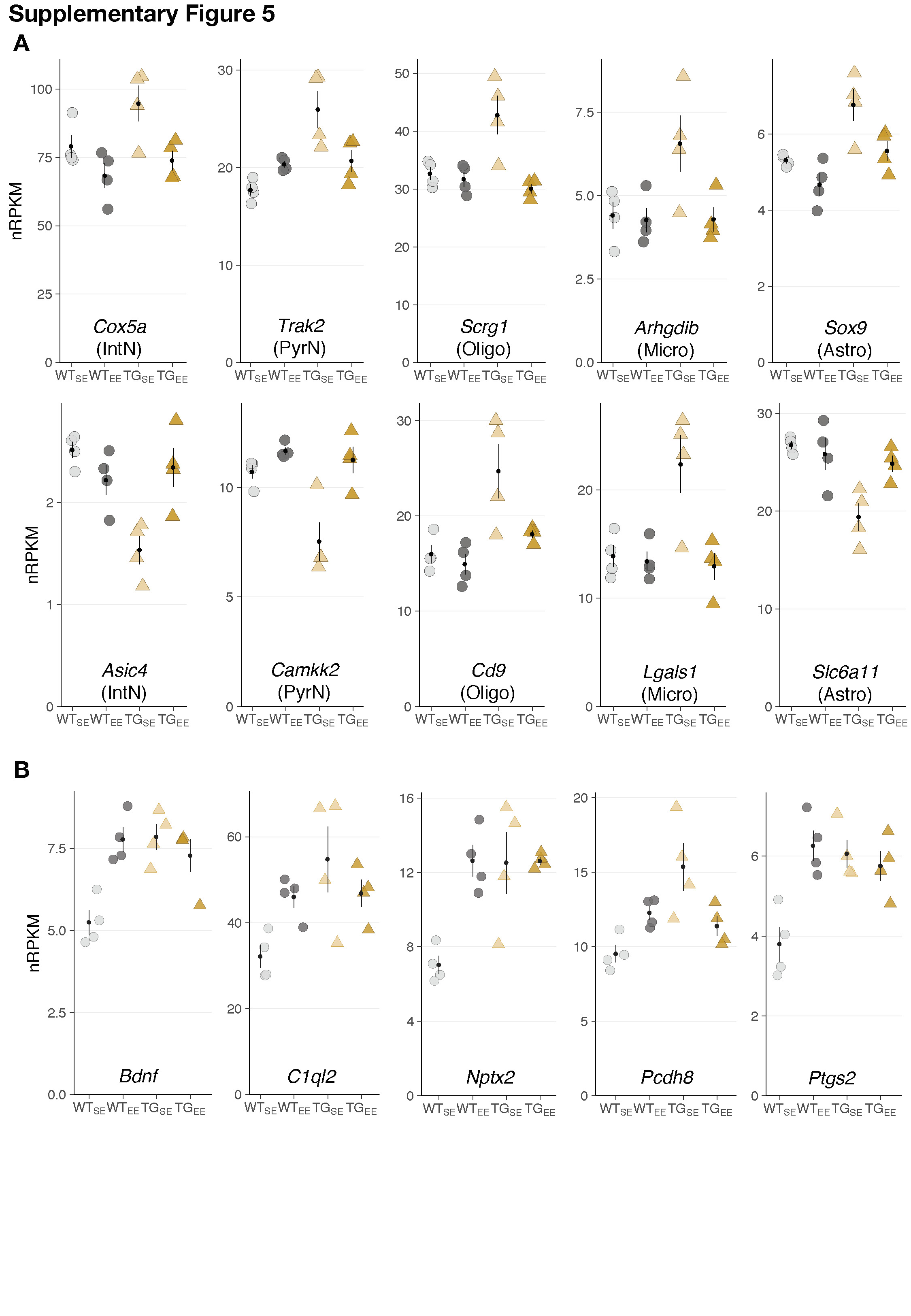

Supplement: Supplementary Figure 5 — Two primary response types to the environmental enrichment in the context of SNCA-induced alterations. (A) Expression levels of selected genes that were prevented from expression changes and showed near control expression levels in TGEE plotted as individual data point with mean ± SEM. (B) Expression levels of selected genes with a similar response to the transgene and the EE plotted as individual data point with mean ± SEM. [file Image_5.jpeg]

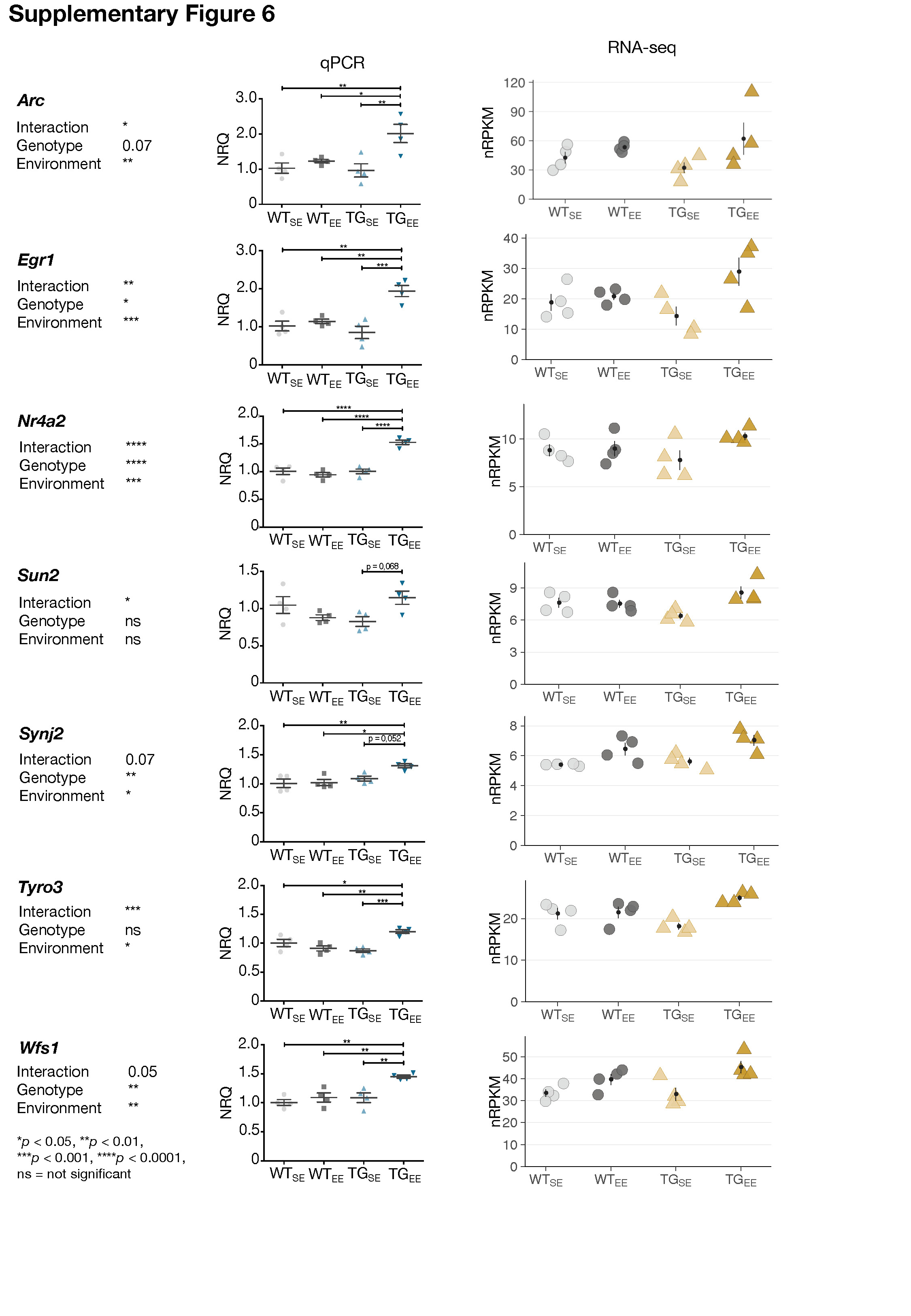

Supplement: Supplementary Figure 6 — Validation of RNA-seq results using RT-qPCR. RNA sequencing results of selected targets identified in Cluster VI and VII (Figure 4) were verified by RT-qPCR. (Left) shows RT-qPCR normalized quantities relative to WTSE (individual data points with mean ± SEM). (Right) Shows expression levels in normalized reads per kilobase per million (nRPKMs) as individual data points with mean ± SEM based on RNA-seq data. For RT-qPCR data, two-way ANOVA with Tukey's post hoc test was performed. [file Image_6.jpeg]

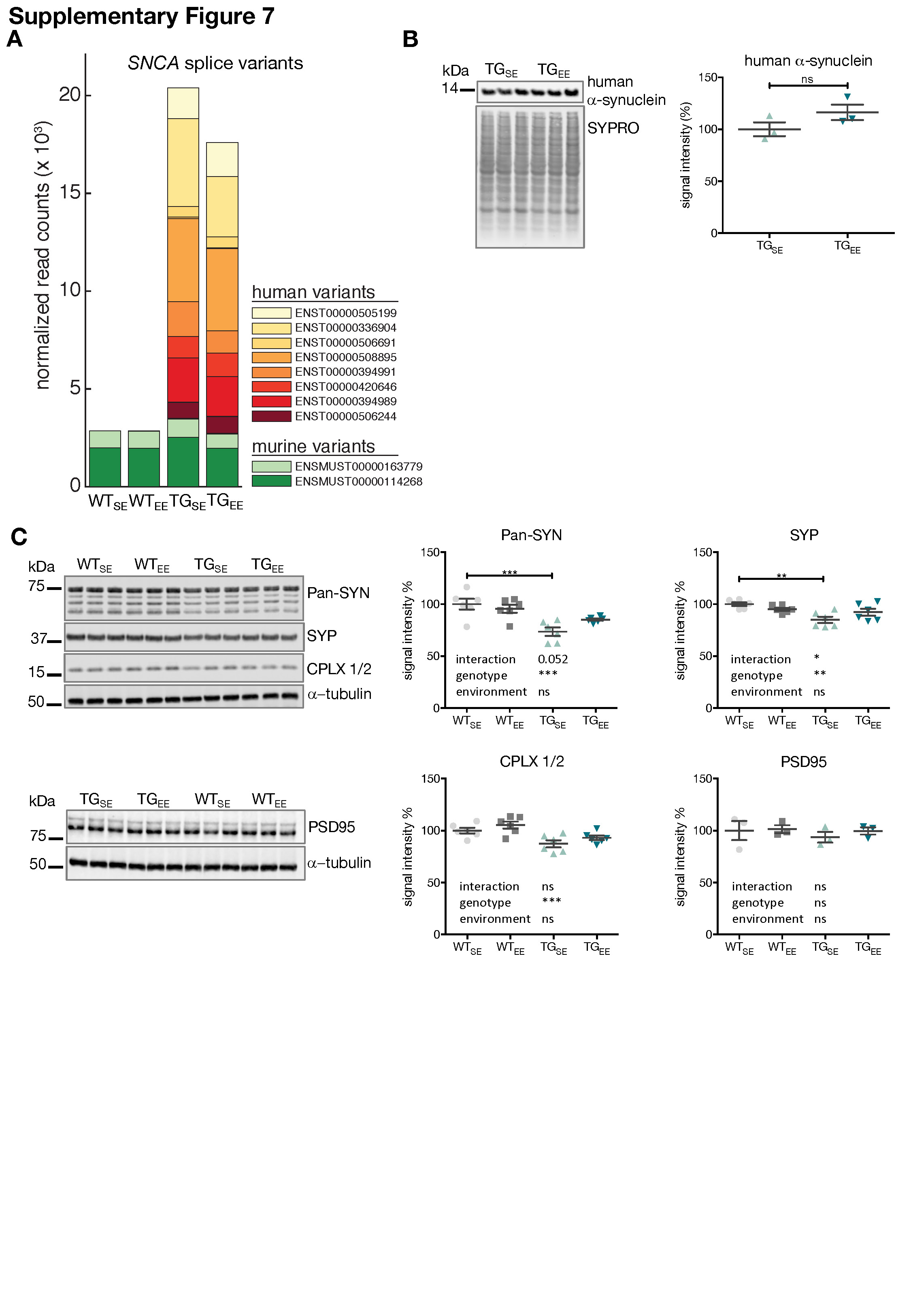

Supplement: Supplementary Figure 7 — Alpha-synuclein load was unaffected by environmental enrichment. (A) Composition and expression level of murine and human SNCA splice variants across experimental groups. (B) Representative protein blot of human alpha-synuclein protein levels in hippocampal lysates of 12-month-old TGSE and TGEE mice. SYPRO protein blot stain used for normalization (n = 3, unpaired t-test, ns = not significant). (C) Representative protein blots of pre- and post-synaptic protein levels detected in hippocampal lysates across experimental groups (n = 3–6 mice per group). alpha-tubulin was used for normalization. Graph shows quantification relative to WTSE plotted as mean ± SEM. Two-way ANOVA followed by Tukey's multiple comparisons test was performed. *p < 0.05, **p < 0.01, ***p < 0.001, ns = not significant. Note different loading order between blots. See Supplementary Table 2 for details. CS, citrate synthase; SYP, synaptophysin; Pan-SYN, pan-synapsin; CPLX, complexin. [file Image_7.jpeg]

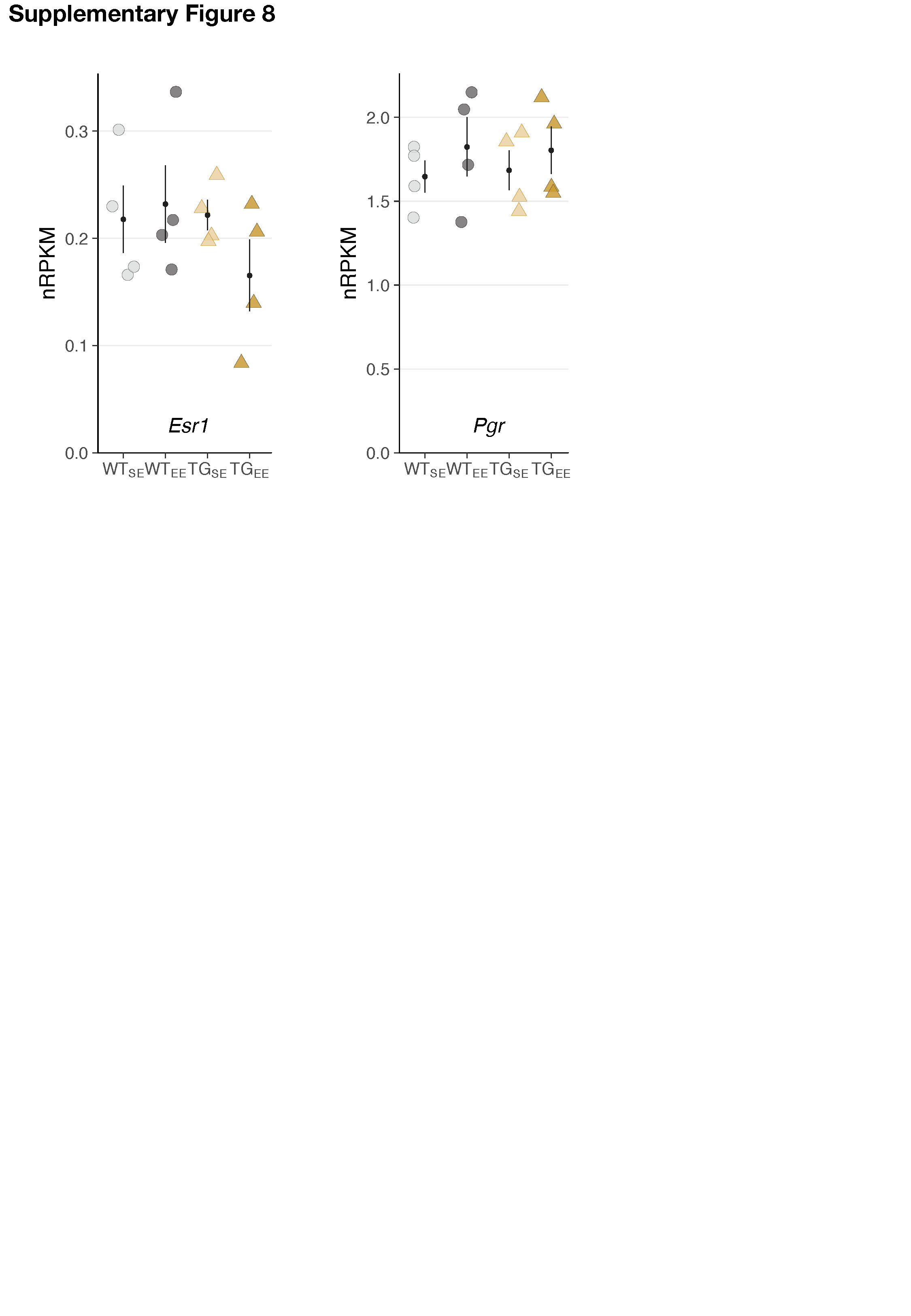

Supplement: Supplementary file 9 [file Image_8.jpeg]
