# Supplementary material for: Environmental Enrichment Prevents Transcriptional Disturbances Induced by Alpha-Synuclein Overexpression
Source: Front Cell Neurosci. 2018 Apr 24;12:112. doi: 10.3389/fncel.2018.00112 (PMC5932345; doi:10.3389/fncel.2018.00112)
Supplement: Supplementary Table 1 — Primer list. Primers used for RT-qPCR validation of RNA-sequencing, along with genotyping primers used in PCR and qPCR assays. [file Data_Sheet_1.docx]

## Supplementary Table 1. Primer list

Primers used for RT-qPCR validation of RNA-sequencing, along with genotyping primers used in PCR and qPCR assays.

## Supplementary Table 2. Comprehensive two-way ANOVA test results

Two-way ANOVA results for protein and RT-qPCR assays.

| **Supplementary Table1**  **Primers used for RT-qPCR validation of gene expression** | |  |
| --- | --- | --- |
|  |  |  |
| **gene** | **sequence 5'-3'** |  |
| Nurr1 (Nr4a2)_f | TCAGAGCCCACGTCGATT |  |
| Nurr1 (Nr4a2)_r | TAGTCAGGGTTTGCCTGGAA |  |
| Egr1_f | CACCTGACCACAGAGTCCTTTT |  |
| Egr1_r | GGGAGAAGCGGCCAGTATAG |  |
| Arc_f | TACCAGTGAAGAAGAGCAGAGC |  |
| Arc_r | GCAGCCTTGAGACCTGGTG |  |
| Homer1a_f | GAAGTCGCAGGAGAAGATG |  |
| Homer1a_r | TGATTGCTGAATTGAATGTGTACC |  |
| Homer1b/c_f | AGGCAACAATGCCAAACTCAC |  |
| Homer1b/c_r | AGCTCAGTGACCCGCTTG |  |
| Wfs1_f | CAGAGCTGGTCCCATGAAGG |  |
| Wfs1_r | AGGCGTAGGTAGTGTTTGCC |  |
| Tyro3_f | AGGCCACATTGGATAGCCTG |  |
| Tyro3_r | CTTCCTGCTTTAGCTGGGCT |  |
| Gfap_f | CCTGAGGCAGAAGCTCCAAG |  |
| Gfap_r | TCTCCTCCTCCAGCGATTCA |  |
| Sdha_f | GCAGCACAGGGAGGTATCA |  |
| Sdha_r | CTCAACCACAGAGGCAGGA |  |
| Tfrc_f | AGGTTGCAAATGCCCAAAGC |  |
| Tfrc_r | GATGAGCATGTCCAAAGAGTGC |  |
|  |  |  |
| **Primers used for genotyping** | |  |
| **gene** | **sequence 5'-3'** | **application** |
| hSNCA_f | CCGCTCGAGCGGTAGGACCGCTTGTTTTAGAC | qPCR |
| hSNCA_r | CCTCTTTCCACGCCACTATC | qPCR |
| hSNCA_f | GTAAAACGACGGCCAGTGCCCCGAAAGTTCTCATTCAA | PCR |
| hSNCA_r | GGAAACAGCTATGACCATGACCCATCACTCATGAACAAGC | PCR |

| **Supplementary Table2** | | | | | | | | | | | | | | | |  | | | |  |
| --- | --- | --- | --- | --- | --- | --- | --- | --- | --- | --- | --- | --- | --- | --- | --- | --- | --- | --- | --- | --- |
| **Protein analyses** | | | | | | | | | | | | | | | |  | | | |  |
| **Two-way ANOVA results** | | | | | | | | | |  | | |  | | |  | | | |  |
|  |  |  |  |  | |  | |  | | | |  | | |  | | |  |  |  |
| **Protein name** | **Alpha** **=** **0.05 n** **=** **3-6** | | | | | | | | | | | | | | | | | | |  |
|  | **Genotype** | | | **Environment** | | | | | **Interaction** | | | | | | | | | |  |  |
| GFAP | F (1, 20) = 7.86, p = 0.0110 | | | F (1, 20) = 7.12, p = 0.0148 | | | | | F (1, 20) = 1.09, p = 0.3088 | | | | | | | | | |  |  |
| EGR1 | F (1, 8) = 5.98, p = 0.0402 | | | F (1, 8) = 0.11, p = 0.7441 | | | | | F (1, 8) = 6.84, p = 0.0309 | | | | | | | | | |  |  |
| NURR1 | F (1, 20) = 51.27, p < 0.0001 | | | F (1, 20) = 3.68, p = 0.0694 | | | | | F (1, 20) = 5.31, p = 0.0321 | | | | | | | | | |  |  |
| SYP | F (1, 20) = 13.07, p = 0.0017 | | | F (1, 20) = 0.25, p = 0.6240 | | | | | F (1, 20) = 6.04, p = 0.0233 | | | | | | | | | |  |  |
| PSD95 | F (1, 8) = 0.48, p = 0.5070 | | | F (1, 8) = 0.42, p = 0.5374 | | | | | F (1, 8) = 0.17, p = 0.6929 | | | | | | | | | |  |  |
| Pan-SYN | F (1, 20) = 22.85, p = 0.0001 | | | F (1, 20) = 0.89, p = 0.3582 | | | | | F (1, 20) = 4.25, p = 0.0524 | | | | | | | | | |  |  |
| CPLX | F (1, 20) = 17.86, p = 0.0004 | | | F (1, 20) = 3.53, p = 0.0749 | | | | | F (1, 20) = 0.01, p = 0.9334 | | | | | | | | | |  |  |
|  |  |  |  |  |  | |  | | | |  | | |  | | |  | | | |
| **Protein name** | **Alpha** **=** **0.05 n** **=** **3-6** | | | | | | | | | | | | | | | | | | |  |
|  | **Genotype** | | | **Age** | | | | | **Interaction** | | | | | | | | | |  |  |
| Pan-SYN | F (1, 20) = 21.82, p = 0.0001 | | | F (1, 20) = 4.13, p = 0.0556 | | | | | F (1, 20) = 0.15, p = 0.7077 | | | | | | | | | |  |  |
| SYP | F (1, 20) = 22.59, p = 0.0001 | | | F (1, 20) = 0.25, p = 0.6201 | | | | | F (1, 20) = 0.09, p = 0.7637 | | | | | | | | | |  |  |
| VAMP | F (1, 8) = 2.10, p = 0.1849 | | | F (1, 8) = 3.26, p = 0.1087 | | | | | F (1, 8) = 5.38, p = 0.0489 | | | | | | | | | |  |  |

| **Reverse transcription-quantitative PCR (RT-qPCR)** | | | | |
| --- | --- | --- | --- | --- |
| **Two-way ANOVA results** | | | | |
|  | | | | |
| **gene name** | **Alpha** **=** **0.05 n** **=** **4** | | |  |
|  | **Genotype** | **Environment** | **Interaction** |  |
| *Gfap* | F (1, 12) = 6.25, p = 0.0279 | F (1, 12) = 4.16, p = 0.0641 | F (1, 12) = 6.04, p = 0.0302 |  |
| *Homer1a* | F (1, 12) = 0.63, p = 0.4419 | F (1, 12) = 12.13, p = 0.0045 | F (1, 12) = 5.96, p = 0.0311 |  |
| *Homer1b/c* | F (1, 12) = 1.29, p = 0.2776 | F (1, 12) = 8.78, p = 0.0119 | F (1, 12) = 2.31, p = 0.1542 |  |
| *Arc* | F (1, 12) = 4.09, p = 0.0661 | F (1, 12) = 12.31, p = 0.0043 | F (1, 12) = 5.69, p = 0.0344 |  |
| *Egr1* | F (1, 12) = 5.80, p = 0.0330 | F (1, 12) = 21.39, p = 0.0006 | F (1, 12) = 13.61, p = 0.0031 |  |
| *Nr4a2* (*Nurr1*) | F (1, 12) = 38.60, p < 0.0001 | F (1, 12) = 24.34, p = 0.0003 | F (1, 12) = 39.27, p < 0.0001 |  |
| *Tyro3* | F (1, 12) = 3.22, p = 0.0978 | F (1, 12) = 7.05, p = 0.0210 | F (1, 12) = 23.27, p = 0.0004 |  |
| *Wfs1* | F (1, 12) = 11.79, p = 0.0050 | F (1, 12) = 12.35, p = 0.0043 | F (1, 12) = 4.69, p = 0.0513 |  |
